# MicroRNA Profiling as Tool for *In Vitro* Developmental Neurotoxicity Testing: The Case of Sodium Valproate

**DOI:** 10.1371/journal.pone.0098892

**Published:** 2014-06-04

**Authors:** Lena Smirnova, Katharina Block, Alexandra Sittka, Michael Oelgeschläger, Andrea E. M. Seiler, Andreas Luch

**Affiliations:** Federal Institute for Risk Assessment (BfR), Berlin, Germany; Stem Cell Research Institute, Belgium

## Abstract

Studying chemical disturbances during neural differentiation of murine embryonic stem cells (mESCs) has been established as an alternative *in vitro* testing approach for the identification of developmental neurotoxicants. miRNAs represent a class of small non-coding RNA molecules involved in the regulation of neural development and ESC differentiation and specification. Thus, neural differentiation of mESCs *in vitro* allows investigating the role of miRNAs in chemical-mediated developmental toxicity. We analyzed changes in miRNome and transcriptome during neural differentiation of mESCs exposed to the developmental neurotoxicant sodium valproate (VPA). A total of 110 miRNAs and 377 mRNAs were identified differently expressed in neurally differentiating mESCs upon VPA treatment. Based on miRNA profiling we observed that VPA shifts the lineage specification from neural to myogenic differentiation (upregulation of muscle-abundant miRNAs, *mir-206, mir-133a* and *mir-10a*, and downregulation of neural-specific *mir-124a, mir-128* and *mir-137*). These findings were confirmed on the mRNA level and via immunochemistry. Particularly, the expression of myogenic regulatory factors (MRFs) as well as muscle-specific genes (*Actc1, calponin*, *myosin light chain, asporin, decorin*) were found elevated, while genes involved in neurogenesis (e.g. *Otx1*, *2, and Zic3, 4, 5*) were repressed. These results were specific for valproate treatment and―based on the following two observations―most likely due to the inhibition of histone deacetylase (HDAC) activity: (i) we did not observe any induction of muscle-specific miRNAs in neurally differentiating mESCs exposed to the unrelated developmental neurotoxicant sodium arsenite; and (ii) the expression of muscle-abundant *mir-206* and *mir-10a* was similarly increased in cells exposed to the structurally different HDAC inhibitor trichostatin A (TSA). Based on our results we conclude that miRNA expression profiling is a suitable molecular endpoint for developmental neurotoxicity. The observed lineage shift into myogenesis, where miRNAs may play an important role, could be one of the developmental neurotoxic mechanisms of VPA.

## Introduction

Exposures to xenobiotics during embryonic, fetal, and perinatal periods are of particular concern since scientific evidence suggest that the developing central nervous system (CNS) is much more vulnerable to chemicals than the adult CNS. Exposure to drugs and environmental chemicals during critical developmental stages is likely to contribute to the increasing incidence of neurodevelopmental disorders in children [1]–[5]. Today one out of six children is diagnosed with a developmental disorder [6] [7], which include, for instance, learning disabilities and delays, autism spectrum disorders (ASD), and the attention deficit and hyperactivity disorder (ADHD). There is a critical deficiency of knowledge when it comes to the developmental neurotoxicity of drugs and chemicals. So far, only very few compounds have been identified as developmental neurotoxicants [1], but this might not reflect the actual prevalence of neurotoxicants in the human environment, since only a minor portion of the more than 80,000 chemicals used worldwide have been tested to determine their potential to trigger developmental neurotoxicity (DNT) *in vivo*. An important restriction for routine assessment of chemical-mediated DNT is the high cost–approximately $1.4 million per substance–and the time consumption associated with the conduct of regulatory *in vivo* tests currently accepted at the level of international guidelines (OECD TG 426 and US EPA 712-C-98-239) [8] [9]. Hence, there is a critical need for the development of alternative non-animal, high-throughput methods for DNT assessment to ensure the safety of chemicals and drugs.

Several *in vitro* DNT approaches addressing different aspects of neurogenesis have been developed during the last two decades. These include studies on invertebrates such as nematodes (*Caenorhabditis elegans*) or flies (*Drosophila melanogaster*), vertebrate models such as zebrafish, as well as different cellular systems such as mouse (m) or human (h) embryonic stem cells (ESCs), neural progenitor cells (NPCs), or primary cell cultures (reviewed in [10]). We prioritized the use of pluripotent mESC-based approaches since differentiation of ESCs covers early stages of neurogenesis, which are not represented in NPCs or primary cells. The protocol for neural differentiation of mESCs has been previously developed and successfully adapted for chemical exposure [11]. This simple, robust, and reproducible method of neural differentiation of mESC is a suitable cellular model to study molecular perturbations induced by chemical exposure during neurogenesis that might provide an insight into the underlying molecular mechanisms and pathways mediating the toxicity of DNT substances.

As a toxicological molecular endpoint, microRNA (miRNA) expression profiling was chosen. MiRNAs are small noncoding RNA molecules (21–25 nt), which were discovered a decade ago as powerful post-transriptional regulators of embryonic development [12]. The expression of thousand of genes is regulated by hundreds of different miRNAs that bind to (imperfect) complementarity sites in the 3′UTR regions of the respective target mRNAs, thereby interfering with the translation of the encoded proteins [13]. One miRNA may have several hundreds of targets and one target mRNA may be regulated by more than one miRNA. More then 60% of the protein coding genes are predicted to be regulated by miRNAs [14]. Increasing evidence points to the importance and significance of miRNA networks in coordination and fine-tuning of gene expression with high temporal and spatial specificity (reviewed in [15]). More than 50% of all identified miRNAs are expressed in the brain, where they play a particularly significant role in brain development by regulating developmental timing, cellular differentiation, proliferation, lineage determination, synaptogenesis, and brain morphogenesis (reviewed in [16] and [17]). In animal studies, depletion of the RNAse III Dicer triggers the loss of miRNA synthesis and results in severe defects in brain development and morphogenesis [18], [19]. Taking into account both the significant role of miRNA in the development of the CNS, and the fact that mRNAs with miRNA binding sites are twice as likely to be sensitive to environmental chemicals exposure than those which lack miRNA binding sites [20], suggests that miRNA (with their ability to post-transcriptionally regulate thousands of genes) can rapidly respond to environmental disturbances. Moreover, many miRNA are phylogenetically conserved molecules, which makes them perfect endpoints in toxicological studies. Studying the perturbations of phylogenetically conserved miRNAs may help to overcome the problem of interspecies differences and allow the extrapolation of the results obtained in non-human species to humans. However, the role of miRNAs in toxicology, especially neurotoxicology, still remains in an exploratory phase. Several studies have addressed the question of the role and possible consequences of perturbations in miRNA expression under exposure to environmental toxicants or drugs (reviewed in [21]–[23]). Transcriptomics perturbations during neural differentiation of mESC induced by developmental neurotoxicants were extensively studied by Piersma's group [24], [25]. They did not, however, address the question of miRNA profiling. Recently, miRNA profiling as a toll for DNT was assed by Bal-Price's laboratory with methyl mercury chloride as the example [26].

In this study we analyzed miRNA expression profiles (miRNome) after neural differentiation of mESCs under the exposure to a well known developmental neurotoxicant: sodium valproate (salt of valproic acid, VPA) [27] to establish miRNA profiling as a molecular tool for DNT testing. VPA is a broadly used anti-epileptic drug, and is also applied for the treatment of bipolar disorder, cancer, and migraine [28]–[30]. The therapeutic effect of VPA is a combination of several effects on cellular signalling, including induction of GABAergic neurotransmission, promotion of neuronal remodelling, and neurogenesis [31]–[33]. The teratogenic effects of VPA that result in neural tube defects (NTD), heart malformations, and craniofacial and skeletal anomalies are well described. The mechanisms underlying these malformations, however, remain ambiguous [27]. VPA was shown to influence N-cadherin-mediated cell adhesion, to modulate Wnt-dependent gene expression, and to inhibit histone deacetylase (HDAC) activities [34], thereby facilitating the establishment of a pluripotent state [35]–[37]. Since miRNAs have been shown to represent a major target of HDAC inhibitors (reviewed in [38]), miRNA might also be important components of the molecular mechanisms mediating the neurotoxic effects of VPA. To analyze whether VPA effects on miRNA expression are compound-specific, we also exposed the cells to another epigenetically active developmental neurotoxicant, sodium arsenite. Arsenic is a very toxic, carcinogenic environmental contaminant associated with the induction of neurobehavioral dysfunctions [39], [40]. The results of this study provided new molecular insight into the mechanisms mediating VPA toxicity: long term exposure to valproate but not arsenite during neural differentiation of mES cells promoted disbalance in lineage specification by induction of myogenesis in neuronally differentiating cultures. We conclude that miRNA expression profiling can be used as a novel molecular endpoint for the identification of DNT activities and substance specific effects.

## Materials and Methods

### Cell culture and neural differentiation

mESC line W4 [41], kindly provided by Dr. Rolf Kemler from the Max Planck Institute, Freiburg, Germany, was cultured in high glucose (4.5 g/l) Dulbecco's modified Eagle's medium (DMEM; Gibco Invotrogen) containing 15% PANSERA-ES fetal bovine serum (FBS, PAN Biotech GmbH), 2 mM glutamate, 50 U/ml penicillin, 50 µg/ml streptomycin, 1% nonessential amino acids (Gibco Invitrogen), 0.1 mM β-mercaptoethanol (Sigma), and 1000 U/ml murine leukemia inhibitory factor (mLIF, Chemicon). Cells were maintained feeder-free in humidified atmosphere at 37°C and 5% CO_2_. Cells were routinely sub-cultured every 2-3 days (in a split-ratio 1∶10–1∶20) until passage 25.

Neural differentiation was induced according to the established protocol [11]. Briefly, prior to induction of neural differentiation (day 0), W4 cells were cultured at high density in routine culture media in the presence of mLIF for 24 h. On the next day (day 1), neural differentiation was induced by plating the cells at low density in DMEM-F12/Neurobasal Medium (1∶1), supplemented with B27 (Gibco, 1∶100), N2 (Gibco, 1∶200), insulin (Sigma, 10 µg/ml), bovine serum albumin, fraction V (Sigma, 150 µg/ml), β-mercaptoethanol (Sigma, 0.1 mM), 50 U/ml penicillin, 50 µg/ml streptomycin, 0.2% FCS (from day one to five), 1 µg/ml laminine, from day one to day seven (Sigma), 10 ng/ml basic fibroblast growth factor, from day seven human (bFGF; Strathmann Biotec AG) on poly-L-ornithine(PLO)-coated dishes. The medium was changed on days 5, 7, 9, 12 and 14.

Murine primary cortical neurons were prepared from C57Bl/6 mice pups on day 17 of embryonic development and kindly provided by Prof. Dr. S. Lehnardt. Dr Lehnardt's group has been routinely isolating murine cortical neurons for their experiments and provided us the surplus of the cultures as a gift. The experimental procedure of primary neuron isolation, ethical statement and respective permits are described elsewhere [42], [43]. Neurons were plated at a density of 1.7×10^5^/cm^2^ on poly-D-lysine-coated culture flasks in Neurobasal medium (Gibco) supplemented with 2% B-27 (Gibco), 2 mM glutamine, 50 U/ml penicillin and 50 µg/ml streptomycin.

### Flow cytometry

The efficiency of neural differentiation was estimated by flow cytometry as described in [44]. Briefly, cells were harvested either 12 or 16 days after induction of neural differentiation by trypsin/EDTA treatment, fixed with 2% paraformaldehyde (PFA), permeabilized with 0,15% saponin (Sigma) and stained with anti-βIII-tubulin (1∶3200, Sigma) and anti-MAP2 (1∶800, BD Biosciences) followed by incubation with *R*-phycoerythrin-conjugated goat antiserum (1∶60, DakoCytomation). The cells were analyzed in a BD Calibur flow cytometer (Fig. S1C). Cells lacking primary antibody were used as a control. Expression of stem cell markers Oct3/4 and Nanog (Fig S1A) was analyzed using Human Pluripotent Stem Cells Transcription Factor Analysis Kit according to the manufacturer's instructions (BD).

### Immunocytochemistry

The expression of neural- and muscle-specific markers was determined by indirect immunofluorescence using the following antibodies: anti-βIII-tubulin (1∶3600, Sigma), anti-microtubule-associated protein-2 (MAP2, 1∶1000, BD), anti-α-actinin-sarcomeric (1∶1000, Sigma-Aldrich), anti-nestin (1∶1000, Hybridoma bank-DSHB), anti-GFAP (1∶1600, Chemicon). For immunostaining, cells were plated on PLO-coated chamber slides (Fischer Scientific) and cultured as described above. At appropriate time points cells were fixed in 2% PFA/0.1% Triton X for 20 min at 4°C, washed twice with ice-cold PBS and blocked for 30 min at 4°C (blocking solution: 5% normal goat serum, 1% BSA, 1×PBS). Primary antibodies were diluted in blocking solution and incubated with cells over night at 4°C. After three washes with ice-cold PBS the cells were incubated with Cy2/Cy3-conjugated secondary antibodies (Dianova) for 1 h at room temperature. Nuclei were visualized by staining with the DNA-specific dye 4′,6-diamidino-2-phenylindole (DAPI) for 5 min at 4°C. Slides were photographed using an Olympus BX 51. The staining of mESCs with neural-specific markers on day 9, 12 and 16 after induction of neural differentiation is demonstrated in Fig. S1D. α-Actinin-positive cells were counted manually in three independent experiments using 20-fold magnification. Alkaline phosphatase staining was performed using Alkaline Phosphatase detection kit (Millipore) according to manufacturer's instructions.

### Substance treatment and viability assay

**Figure 1:**
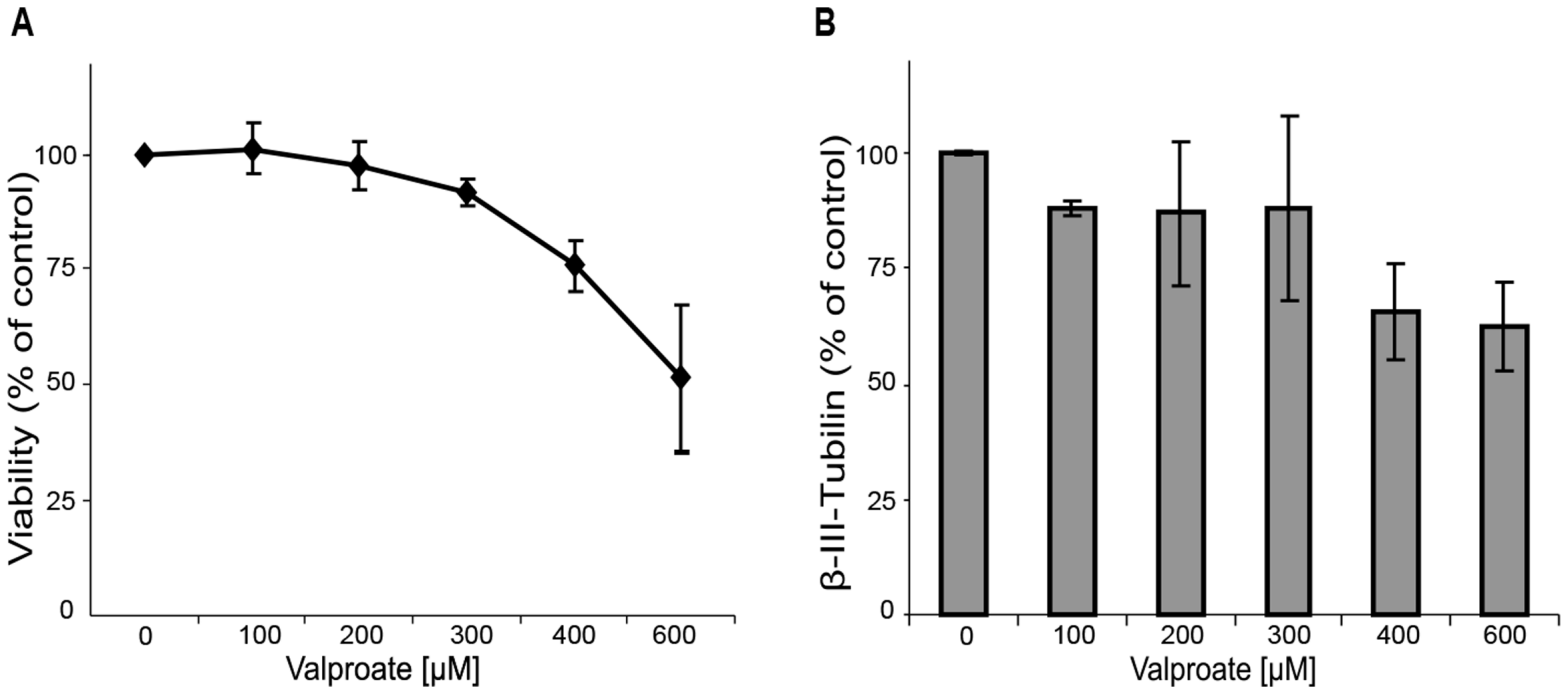
Valproate effects on viability and expression of β-III-Tubulin. The cells were induced to differentiate into neurons for 16 days under continuous substance exposure. Cell viability was estimated using CellTiterBlue assay and is shown as a percentage of solvent control (A), expression of β-III-tubulin was analyzed by flow cytometry and is shown as a percentage of solvent control for each concentration tested (B). Results represent a mean of three independent differentiation experiments ± SEM.

mESCs were exposed from day one of neural differentiation onward to sub-toxic concentrations of the sodium valproate (VPA, Sigma Aldrich), sodium arsenite (As-III, Sigma Aldrich) or trichostatin (TSA, Sigma Aldrich). VPA stock was prepared in PBS, while arsenite and TSA stocks were prepared in water, thus PBS/water controls were included as solvent/untreated controls. The cell culture medium, containing appropriate concentrations of test substances, was exchanged every second day. Cells were harvest on day 0, 5, 7, 9, 12, and 16 of neural differentiation. To estimate the sub-toxic concentrations (IC_10_–C_20_) of test chemicals (VPA, arsenite and TSA) applicable for further gene expression analyses, cell viability was measured using the resazurin dye reduction assay (CellTiterBlue assay, Promega, Fig. 1A and Fig. S4A and B). The effective concentrations that interfere with neural differentiation were estimated using flow cytometry with the neuron-specific marker βIII-tubulin. For microarray analyses cells were treated with 300 µM VPA (IC_10_) or 0.75 µM (IC_10_) arsenite, respectively. For RT-PCR analyses cells were exposed to 200, 300 or 400 µM VPA and to 0.75, 1 or 2 µM TSA, respectively.

Mouse primary embryonic cortical neurons were exposed to higher VPA concentrations than differentiating cultures, since they were found to be less sensitive to the toxicant (observation based on cell viability (resazurin dye reduction) assay, Fig. S4C). Four hours after isolation cells were treated with 0.8 mM, 1 mM and 2 mM VPA for 10 days *in vitro*.

### RNA isolation and reverse transcription

Total RNA was purified from cells using TRIZOL reagent (Invitrogen) from three to four independent differentiation experiments. Isolated RNA was treated with RNAse-free DNAse I (Roche) prior to reverse transcription. For reverse transcription of total RNA, 1.5 µg RNA was reverse-transcribed into single-stranded cDNA using random primers and high capacity cDNA reverse transcription kit (Applied Biosystems) according to manufacturer's instructions. Short stem-loop cDNA libraries from individual miRNAs were generated using TaqMan microRNA assay and TaqMan miRNA reverse transcription kits (Applied Biosystems). For simultaneous detection of multiple miRNAs up to six miRNA-specific stem-loop RT-primers were multiplexed in one reverse transcription reaction. Briefly, In 30 µl reaction 30 ng of total RNA were mixed with 2 µl of each miRNA-specific primer, 3 µl 10× RT Puffer, 0.6 µl dNTP mix (100 mM total), 0.38 µl RNAse inhibitor (20 U/µl) and 2 µl Multiscribe reverse transcriptase (50 U/µl). One µl of cDNA was used for further real-time PCR. cDNA synthesis was performed at 42°C for 30 min.

### miRNA expression microarray profiling

Sample preparation, microarray hybridization and scanning were performed at MFT Services (Faculty of Medicine, University of Tübingen). Briefly, one microgram of RNA, isolated on day 16 of neural differentiation from cells treated either with solvent, VPA or arsenite, was labeled with FlashTag Biotin RNA labeling kit (Genisphere, Hatfield, PA, USA) for Affymetrix GeneChip miRNA 2.0 arrays (Affymetrix, Santa Clara, CA, USA) according to the manufacturer's recommendations. A simple colorimetric Enzyme Linked Oligosorbent Assay (ELOSA) was used to confirm successful biotin labeling. After labeling, the samples were hybridized on Affymetrix GeneChip miRNA 2.0 arrays, washed, stained, and scanned according to manufacturer's instructions (Affymetrix). The data files (.CEL) were imported to Partek 6.5 software (www.partek.com). The RMA method was used to perform background adjustment, quantile normalization and summarization of the log-expression values for each gene on each array. We performed a differential expression analysis using *t*-statistics methods implemented in the Partek software. Ingenutity (IPA) software (Ingenuity Systems, www.ingenuity.com) was used to perform miRNA target genes filtering and to identify potential gene networks, pathways and biological functions in which VPA sensitive miRNAs could be involved.

### Whole-genome expression microarray profiling

Total RNA was purified from either VPA-treated or untreated mESCs on day 16 of neural differentiation. Their expression profiles were analyzed with the GeneChip Mouse Gene 1.0 ST Array (Affymetrix). The amount and integrity of purified RNA were estimated by microcapillary electrophoresis (2100 Bioanalyzer, Agilent Technologies), whereas purity was assessed by the *A*260/*A*280 ratio.

Amplified and biotinylated sense strand DNA was generated from total RNA with the Affymetrix whole transcript sense target labeling assay. Labeled, single-stranded DNA (5.5 µg) was combined with hybridization and spike controls and hybridized with the pre-equilibrated Affymetrix GeneChip Mouse Gene 1.0 ST Array for 16–18 h. Following hybridization, arrays were washed and stained with a streptavidin phycoerythrin conjugate using an automated GeneChip Fluidics Station 450. They were then scanned with a GeneChip Scanner 3000 using a 570-nm excitation wavelength laser. Sample preparation, microarray hybridization and scanning were performed at MFT Services (Faculty of Medicine, University of Tübingen). Microarray quality control assessment and data acquisition were performed with the GeneChip Operating Software (Affymetrix). Normalization among the different microarray data files was performed by robust multi-array analysis (RMA) inside the Partek 6.5 software. Unsupervised hierarchical clustering analysis and a principal component analysis were performed with the Partek software to establish non-forced groups of samples. Differential expression was determined by using *t*-statistics. False discovery rates were determined following the Benjamini-Hochberg procedure [45]. Data were expressed as mean ± S.D. Student's *t*-test was done to determine the statistical significance of the pairwise comparisons.

### Gene network analysis

Upregulated and downregulated miRNA together with their reciprocal expressed mRNA targets were further analyzed using IPA software (Ingenuity Systems, www.ingenuity.com). A data set containing gene identifiers and corresponding expression values was uploaded into the application. Each identifier was mapped to its corresponding object in the Ingenuity Knowledge Base. A ≥2-fold cutoff was set to identify molecules whose expression was significantly differentially regulated. These molecules, called network eligible molecules, were overlaid onto a global molecular network developed from information contained in the Ingenuity Knowledge Base. Molecular networks were then algorithmically generated based on their connectivity. The functional analysis identified the biological functions and/or diseases that were most significant affected by the VPA treatment. Right tailed Fisher's exact test was used to calculate a *p*-value determining the probability that each biological function and/or disease assigned to that data set is due to chance alone. Canonical pathways analysis identified the pathways from the IPA library of canonical pathways that were most significant altered by the VPA treatment. The significance of the association between the data set and the canonical pathway was measured in 2 ways: 1) A ratio of the number of molecules from the data set that map to the pathway divided by the total number of molecules that map to the canonical pathway is displayed; 2) Fisher's exact test was used to calculate a *p*-value determining the probability that the association between the genes in the dataset and the canonical pathway is explained by chance alone.

### Quantitative RT-PCR

Quantitative real-time PCR (RT-PCR) on miRNAs was performed using the TaqMan microRNA assay kit in combination with TaqMan universal PCR master mix, no AmpErase UNG (Applied Biosystems) according manufacturer's instructions. Expression of individual miRNAs was normalized to *sno-202* expression. Expression of primary miRNA transcripts or mRNAs was quantified using SYBR Green reagents (Applied Biosystems). Primers for SYBR Green probes were designed using the NCBI Primer-BLAST software (www.ncbi.nlm.nih.gov/tools/primer-blast/) and are listed in Table S1. *18S* ribosomal RNA was used as endogenous control gene. All RT-PCRs were performed in triplicates on Applied Biosystems 7500 Real-Time PCR System with following thermal cycling parameters: miRNA RT-PCR (95°C for 2 min, followed by 40 cycles of 15 s at 95°C and 1 min 60°C); SYBR Green RT-PCR (50°C for 2 min, 95°C for 2 min, followed by 40 cycles of 15 s at 95°C and 1 min 60°C); Melt curve stage was included in SYBR Green reaction (95°C for 15 s, 60°C for 1 min, 95°C for 30 s and 60°C for 15 s). The relative levels of RNA expression in treated samples in comparison to untreated controls were quantified using the comparative CT (2^−ΔΔCT^) method [46]. Data collected from three or four independent differentiation experiments are reported as average log_2_-fold change of independent biological replicates ± SEM. Differences in treated and untreated samples were analyzed for statistical significance using Student's t-test. P–Value<0.05 was denoted on the graphs by *, p<0.01 by **, and p<0.001 by ***.

## Results

### Valproate effects on cell viability and neural differentiation of mESC

mESC line W4 were differentiated into neurons according to an established in-house protocol [11]. The undifferentiated W4 cells showed a typical ESC morphology and expressed alkaline phosphatase as well as Oct3/4 and Nanog (Fig. S1A). Neural differentiation efficiency was monitored using neural-specific markers by western blot (Fig. S1B), flow cytometry (Fig. S1C) and immunocytochemistry (Fig. S1D). Furthermore, expression of neural-specific/enriched miRNAs, neuronal marker (*β-III-tubulin*) and neuro-progenitor marker (*nestin*) during differentiation was analyzed by qRT-PCR. All neural miRNAs as well as neuronal marker *β-III-tubulin* were strongly induced upon induction of differentiation (Fig. S1E and F). The VPA-induced alterations in the neural differentiation process were monitored after exposure to a range of different valproate concentrations for 16 days. To estimate the sub-toxic concentrations of this compound for subsequent gene expression analyses, we determined the effects of VPA on cell viability and neural differentiation using the resazurin reduction assay (CellTiterBlue) and flow cytometry applying the neuron-specific marker β-III-tubulin, respectively. VPA affected cell viability in a concentration-dependent manner (Fig. 1A). The substance concentration that reduced cell viability to 90% (effective concentration 10%, EC_10_) was 314 µM. Expression of βIII-tubulin was reduced up to 65% of control by VPA (Fig. 1B).

### VPA effects on miRNome of neural-differentiated ESC

As a next step, neurally differentiating mESCs were used to determine the effects of valproate on miRNA expression. In these experiments cells were also treated with a structurally and mechanistically unrelated developmental neurotoxicant and epigenetically active substance, sodium arsenite, in order to investigate substance-specific effects on the miRNA expression profile. To monitor effects of VPA and arsenite on the miRNome, mESCs were differentiated into neurons for 16 days under exposure to sub-toxic concentrations of either sodium valproate (300 µM) or sodium arsenite (0.75 µM). The 16-day period was chosen based on analysis of the expression patterns of neural-specific miRNAs during neural differentiation of mESCs. *mir-9* was strongly induced from day 9 of differentiation onwards, while *mir-124* and *mir-128* were induced later on reaching their maximum expression levels both on day 16 ([47] and Fig. S1F). Brain enriched miRNAs such as *mir-125b*, *let-7a,c*, *mir-138* and *mir-181a* were also strongly induced after 16 days of differentiation ([47] and Fig. S1F). We analyzed the global changes in miRNA profiles in VPA-treated cells using an Affymetrix microarray. The normalized data of miRNA and whole genome microarrays are deposited in the GEOarchive (see: http://www.ncbi.nlm.nih.gov/geo/query/acc.cgi?acc=GSE50215, http://www.ncbi.nlm.nih.gov/geo/query/acc.cgi?acc=GSE50216).

**Figure 2:**
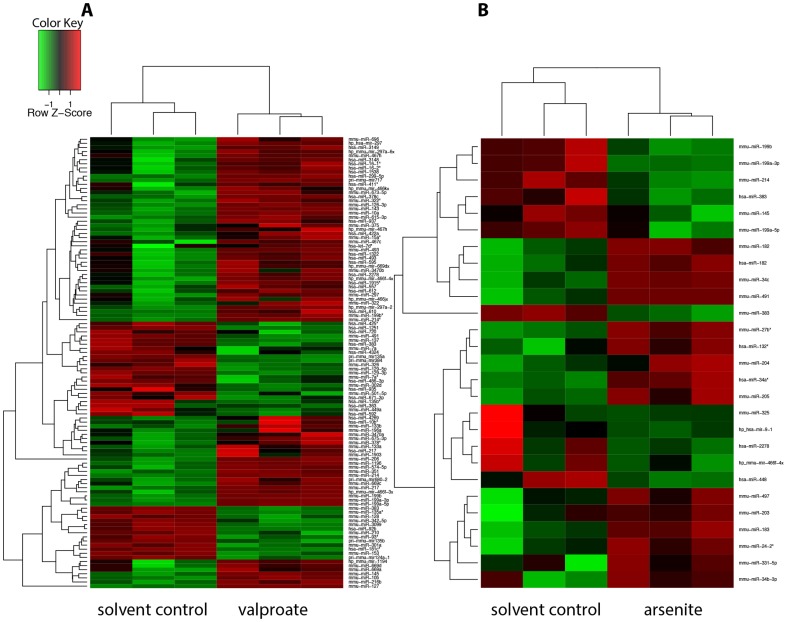
Hierarchical cluster analysis (HCA) of miRNA expression in treated and control samples. HCA was carried out using Euclidian algorithm to build the cluster tree of the average significantly altered miRNAs by VPA or arsenite in comparison to negative/solvent control (NC) in neural- differentiated ESCs. The miRNA expression intensities of all probe set IDs are scaled as a Z-score (all microarray experiments were done in triplicates). Red color denotes upregulated miRNAs, green color – down regulated miRNAs. A. HCA of miRNAs responding to VPA treatment. B. HCA of miRNAs responding to arsenite treatment.

Differently expressed miRNAs are summarized in Fig. 2 and Table S2. The exposure to valproate during neural differentiation of mESCs led to significant alterations (fold change (FC)≥|2|, p≤0.05) in the expression of 110 miRNAs (Fig. 2A).

**Figure 3:**
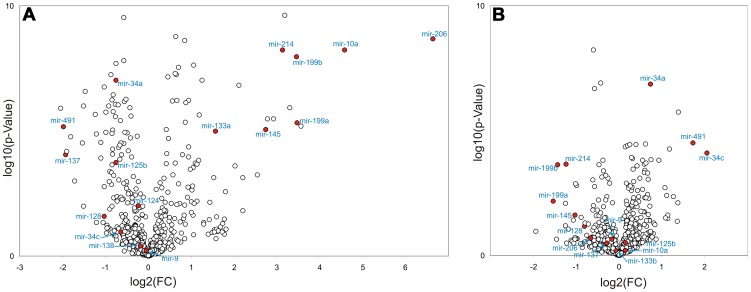
Volcano plots comparing VPA and arsenite miRNA signatures 16 days after neural differentiation of mESCs. The –log_10_ of P-values for each miRNA are plotted against log_2_ mean ratio (three replicates) of the normalized miRNA signals of treated samples compared to solvent control. A. VPA induced changes in miRNA profile of neural-differentiated mESCs. B. Arsenite effects on miRNA expression in neural-induced mESCs. miRNAs which were included in further qPCR analysis or were regulated in opposite direction by both substances are marked in red.

From 110 miRNAs that responded to VPA exposure, 74 were upregulated up to 100-fold, while 36 were 2- to 4-fold downregulated (Table S2). Six miRNAs were regulated on the level of primary transcripts (pri-*mir*_s_) but not on the level of the mature 22-nt forms, as determined via the whole genome array. In contrast, arsenite significantly altered the expression only of 27 miRNAs (Fig. 2B and Table S2). Ten miRNAs were in intersection for both substances, but most of them were regulated in opposite direction (Table S3). From 27 arsenite-regulated miRNAs, expression of 15 miRNAs was significantly increased (up to 4-fold), and the expression of 12 miRNAs was decreased (up to 3-fold) (Table S2). Volcano plots presented in Figure 3 demonstrate substance-specific effects on miRNA expression. The plots represent log_2_ ratios in miRNA expression in substance-treated cells in comparison to control (solvent-treated) cells plotted against −log_10_ of the *p*-value. Comparing to the solvent control, in cells treated with VPA we observed a strong upregulation of myogenic miRNAs (myo-*mir*s: *mir-206, mir-133a,b*), or miRNAs shown to be involved in muscle differentiation and specification (*mir-10a*, *mir-143*/*mir-145 cluster, mir-214, mir-322, mir-199a*). By contrast, we could not observe any induction of muscle-specific miRNAs upon exposure of cells to arsenite. Moreover, *mir-214, mir-145* and *mir-199a* were significantly downregulated in these cells. The myogenic *mir-206* was the most strongly induced miRNA in cells treated with VPA (up to 100-fold increase in expression). However, *mir-133b* that clusters with *mir-206* in a bicistronic loci, was induced by VPA only up to 2.2-fold arguing that the induction of *mir-206* was at least not solely due to a change in chromatin structure.

miRNAs involved in embryonic and adult neurogenesis such as *mir-137*, *mir-128*, *mir-124a*, *mir-326*, or *mir-7* were found significantly downregulated by VPA. No effects of arsenite were observed on the expression of these miRNAs. All of these repressed neural miRNAs were shown to inhibit neural stem cell and neural progenitor cell proliferation and stimulate neuronal differentiation and specification (cf. section Discussion [16]).

Known tumor-suppressor miRNAs, such as *mir-34a* and *c*, were strongly upregulated by arsenite and only slightly downregulated in cells treated with VPA. Further, *mir-491*, a miRNA which is involved in neurosteroidogenesis and pathogenesis of multiple sclerosis [48], was found strongly upregulated by arsenite and downregulated by VPA, and *mir-383*, a miRNA expressed in the reproductive system and a negative regulator of proliferation, was highly expressed in control samples but significantly downregulated by both substances (4.2- and 2-fold by VPA and arsenite, respectively).

Altogether our data demonstrate substance-specific effects on miRNA expression profiles. In particular, long term exposure to valproate, but not arsenite, during neural differentiation of mESCs led to the repression of neural- and the induction of muscle-specific miRNAs.

### VPA effects on protein coding gene expression in neural-differentiated mESCs

**Figure 4:**
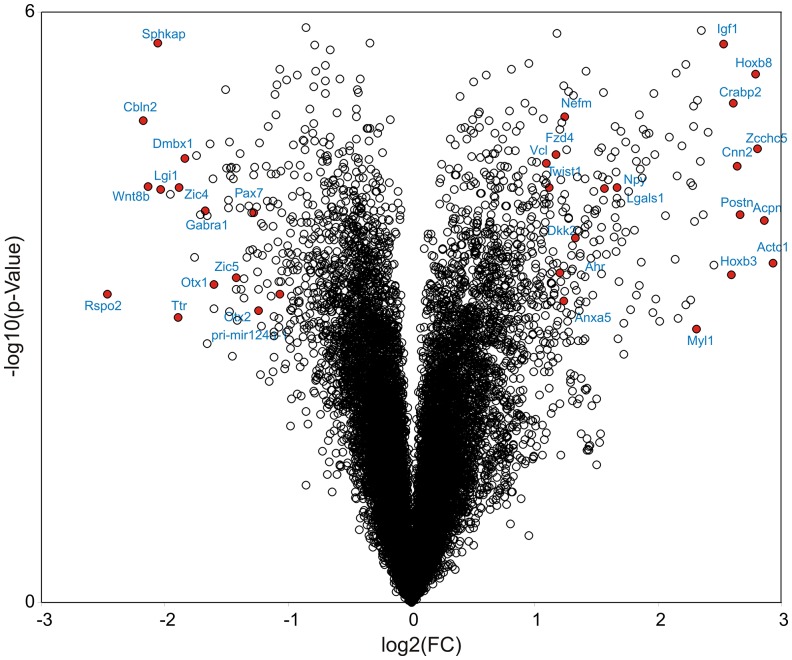
Volcano plots presenting VPA mRNA signature 16 days after neural differentiation of ESCs. The –log_10_ of P-values for each mRNA are plotted against log_2_ mean ratio (from three microarrays) of the normalized mRNA signals of treated samples compared to untreated control.

To support the miRNomics data in cells treated with VPA we performed whole genome microarray analysis using the same RNA samples as for miRNA profiling. Differently expressed genes in cells upon exposure to VPA are summarized in Fig. 4 and Table S4. The expression of 377 genes was significantly affected by VPA (FC>2.0, p<0.05). 267 genes were upregulated up to 7-fold, while 110 genes were 2- to 5-fold downregulated. At the mRNA level, we also could confirm the VPA-mediated induction of myogenesis in cells through the detection of upregulation of the respective genes *Actc1* (most strongly induced by 7.6-fold), *calponin*, *myosin light chain, asporin, and decorin*. In addition, a number of *Hox* genes, including *HoxB2,3,4,5,7,8,9; HoxA2,3; HoxC4,5,6,8 and HoxD4,8*, which are known to be involved in cell fate determination during embryonic development, the regulation of the anterior-posterior polarity, in particular, were strongly upregulated by VPA treatment. Finally, *Twist1*, a transcription factor involved in cell lineage determination and specification, skeletal and muscle system morphogenesis, and strongly expressed in mesodermal derived tissues, was 2.1-fold upregulated by VPA. *Twist1* is also a predicted target of *mir-137* and *mir-363*, both of which were repressed in their expression by VPA (cf. above).

In contrast, neural factors like *Otx1* and *Otx2*, known regulators of forebrain development [49] as well as members of ZIC protein family (*Zic3, 4*, and *5*), were downregulated in cells upon exposure to VPA. *Zic family* is also involved in neural differentiation, forebrain development, and neural tube closure [50]. *Otx2* and *Zic4* are predicted targets of *mir-206*, while *Otx1* and *Zic3* are predicted targets of *mir-199a* and the muscle-specific *mir-133a*, respectively. Thus, the expression of protein encoding genes strongly correlated with the obtained miRNA results, confirming the induction of mesodermal and the repression of neural marker genes as well as a reciprocal expression of mRNAs in respect to the corresponding inhibitory miRNAs.

Some components of the Wnt/β-catenin signaling pathway, including the ligand *Wnt8b, the Wnt-receptor Frizzled homolog 4 (Fzd4)*, regulatory proteins like Dickkopf (*Dkk2*,*3*), secreted frizzled-related protein 2 (*SFRP2*), as well as the beta-catenin interacting proteins, including cell adhesion molecules of the *cadherin (Cdh5,12,19)* and *sox* family *(Sox14)*, were also perturbed by VPA.

In addition, we observed changes in the expression of previously described VPA-sensitive genes also in our cell system (*vinculin* (*Vcl*), *annexin 5* (*Anax5*), *galectin-1 (Lgals1)*, *transgelin 2* (*Tagln2*) [51]). Several of VPA-sensitive genes, shown to be involved in neural tube closure and/or neural tube defects [52], were perturbed in our cell system: *Vcl, Twist1, ZIc5, Tagln2, Lgals1, Anax5, Crebbp, brachyury*, and *Hox* genes. The volcano plot in Figure 4 demonstrates the distribution of VPA-sensitive genes.

### Pathway and functional analysis of miRNA targets regulated by VPA

We performed a network and functional analysis of differently expressed genes using Ingenuity (IPA) software. As a first step of data analysis, we comprised a date-set by applying an IPA miRNA target filter. From 377 VPA-sensitive genes, 186 were found to be predicted or experimentally validated targets of 48 VPA-regulated miRNAs. From those 186 transcripts, 136 were in reciprocal expression patterns with 40 miRNAs (Table S5). As an increasing number of studies demonstrate not only a post-transcriptional regulation by miRNAs but also the reduction in the expression of target mRNAs, for subsequent biofunctional analysis using IPA we only included miRNA targets, which exert reciprocal expression patterns to corresponding miRNAs (cf. material and methods). The functional and network analysis of VPA-sensitive miRNA and their targets demonstrated that they are involved in crucial cell signaling pathways and bioprocesses. The most relevant biological and disease processes as well as cell signaling and metabolic pathways are summarized in Table 1. Canonical pathway analysis identified the pathways from the IPA library that were most significant to VPA-sensitive miRNA and target genes.

**Table 1:** List of biological functions and canonical pathways affected by VPA according to IPA analysis.

Bio Functions	# of perturbed molecules	p-value
Cancer	100	3.26E-20–2.70E-03
Genetic disorder	80	5.68E-10–1.73E-05
Tissue development	79	1.14E-15–2.38E-03
Cellular growth and proliferation	73	7.07E-09–2.30E-03
Cellular development	65	1.35E-08–2.30E-03
Cell death	61	2.96E-06–2.46E-03
Embryonic development	61	8.70E-13–2.38E-03
Skeletal and muscular system development and function	57	5.30E-14–2.38E-03
Organ development	55	8.70E-13–2.30E-03

Canonical pathways	# of regulated molecules in the pathway	- log(p-value)
Hepatic Fibrosis/Hepatic Stellate Cell Activation	11/147 (7%) **↑**	7,14E00
RhoA Signaling	7/114 (6%) **↑**	4,07E00
Axonal Guidance Signaling	12/430 (3%) **↑↓**	3,5E00
Role of Osteoblasts in Rheumatoid Arthritis	8/239 (3%) **↑**	2,95E00
RhoGDI Signaling	7/199 (4%) **↑**	2,84E00
FAK Signaling	5/102 (5%) **↑**	2,83E00
Leukocyte Extravasation Signaling	7/199 (4%) **↑**	2,63E00
Paxillin Signaling	5/112 (4%) **↑**	2,61E00
Integrin Signaling	7/210 (3%) **↑**	2,59E00
G-Protein Coupled Receptor Signaling	12/526 (2%) **↑↓**	2,5E00
Tight Junction Signaling	6/163 (4%) **↑**	2,49E00
PTEN Signaling	5/124 (4%) **↑**	2,45E00
Agrin Interactions at Neuromuscular Junction	4/69 (6%) **↑**	2,42E00
Wnt/β-catenin Signaling	6/172 (3%) **↓**	2,31E00
Caveolar-mediated Endocytosis Signaling	4/85 (5%) **↑**	2,27E00
Human Embryonic Stem Cell Pluripotency	5/150 (3%) **↑**	2,1E00
Clathrin-mediated Endocytosis Signaling	6/195 (3%) **↑**	2,1E00
VEGF Signaling	4/99 (4%) **↑**	1,96E00
GABA Receptor Signaling	3/57 (0%) **↓**	1,89E00
Glioma Signaling	4/112 (4%) **↑**	1,86E00
Glioblastoma Multiforme Signaling	5/168 (3%) **↑**	1,83E00

*Arrows show VPA induced up or down regulation of genes involved in the giving pathway.

Top molecular interactions of VPA regulated miRNAs and their targets are presented in Fig. S2. Molecules connected by the first network (A) were shown to be involved in cancer, cardiovascular, and reproductive diseases. The second network (B) combined molecules, which are involved in skeletal and muscle system development and functions, as well as embryonic and organism development that correlate to the upregulation of muscle specific miRNAs and genes. Further analysis of networks between miRNAs and their reciprocally expressed targets and the experimental validation of miRNA-mRNA regulations may further contribute to the understanding of pathways of VPA toxicity.

### Real-time RT-PCR validation of VPA-responsive miRNAs

**Figure 5:**
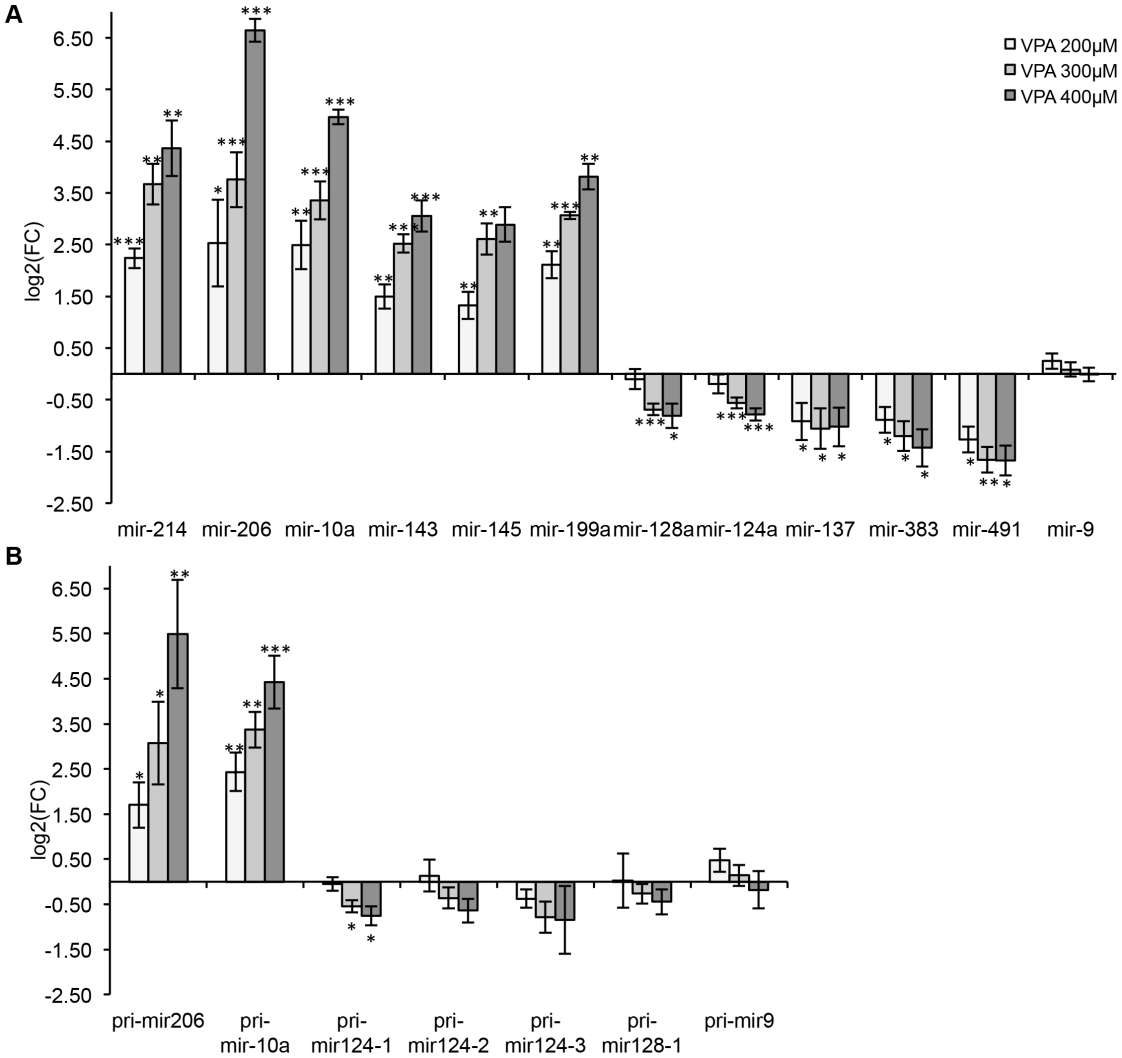
Real-Time PCR verification of Affymetrix miRNA microarray data. A. Expression of mature miRNAs. The graph demonstrates mean of log_2_ fold change (VPA vs. solvent control) ± SEM for six upregulated and five downregulated miRNAs in four independent differentiation processes. *mir-9* expression was not changed significantly upon 16 days of VPA treatment. (n = 4, t-test, *p<0.05, **p<0.01, ***p<0.001). B. Expression of primary miRNA transcripts in neural-differentiated mES cells at day 16 of differentiation under VPA exposure. The graph demonstrates mean of log_2_ fold change from three independent experiments (VPA vs. solvent control) ± SEM. (n = 3, t-test, *p<0.05, **p<0.01, ***p<0.001).

We validated the microarray data using real-time RT-PCR. Taqman microRNA assay (ABI) was used to analyze the expression of a set of muscle- and neuro-specific miRNAs 16 days after induction of neural differentiation under VPA exposure. In four independent differentiation procedures we could confirm the microarray data (Fig. 5A)–that is, a strong concentration-dependent induction of muscle-specific/abundant miRNA (*mir-206, mir-10a, mir-214, mir-145, mir-143, mir-199a*) and a significant downregulation of the expression of neuro-specific miRNAs (*mir-124, mir-128, mir-137, mir-491, mir-383*) in comparison to the solvent control. We did not observe any changes in the expression of neuro-specific *mir-9* in our microarray data, so this miRNA was included in RT-PCR analysis as a negative control. In addition, we analyzed the expression of the same pool of miRNAs in primary cortical neurons, isolated on day 17 of embryonic development from mouse fetus and cultivated for further 10 days under VPA exposure. Only *mir-10a* was significantly affected by VPA in primary cortical neurons, suggesting that this miRNA could be a primary target of VPA (Fig. S3). We could not observe any induction of *mir-206*, or a significant reduction of neuron-specific miRNAs.

Taking into consideration that miRNA expression can be regulated on the level of processing [53], we analyzed the expression of primary transcripts of selected miRNAs as well (Fig. 5B). Both, pri*-mir-10* and pri*-mir-206* were significantly upregulated upon VPA treatment, suggesting that VPA affects the transcription and not the processing of these two miRNAs. Taking into account the known mechanism of VPA as a HDAC inhibitor we can suggest possible activation of *mir-10a* and *mir-206* genes by VPA through the inhibition of HDAC and, as a result, acetylation/activation of histones in the miRNA promoter regions. This would be in line with the induction of *mir-10a* expression in primary neurons (Fig. S3). From two primary *mir-128* transcripts only pri*-mir128-1* was detectable and slightly downregulated in our cell system. From three known primary *mir-124* transcripts pri*-mir124-1* was most strongly upregulated 16 days after induction of neural differentiation and was significantly downregulated by VPA. The reduction in the expression of pri*-mir-124-2* and *3* was not statistically significant. As expected, the substance did not affect the expression of pri*-mir-9*.

### Real-time RT-PCR validation of VPA-responsive mRNAs: Time kinetics of myogenesis and miRNA involved in myogenesis

**Figure 6:**
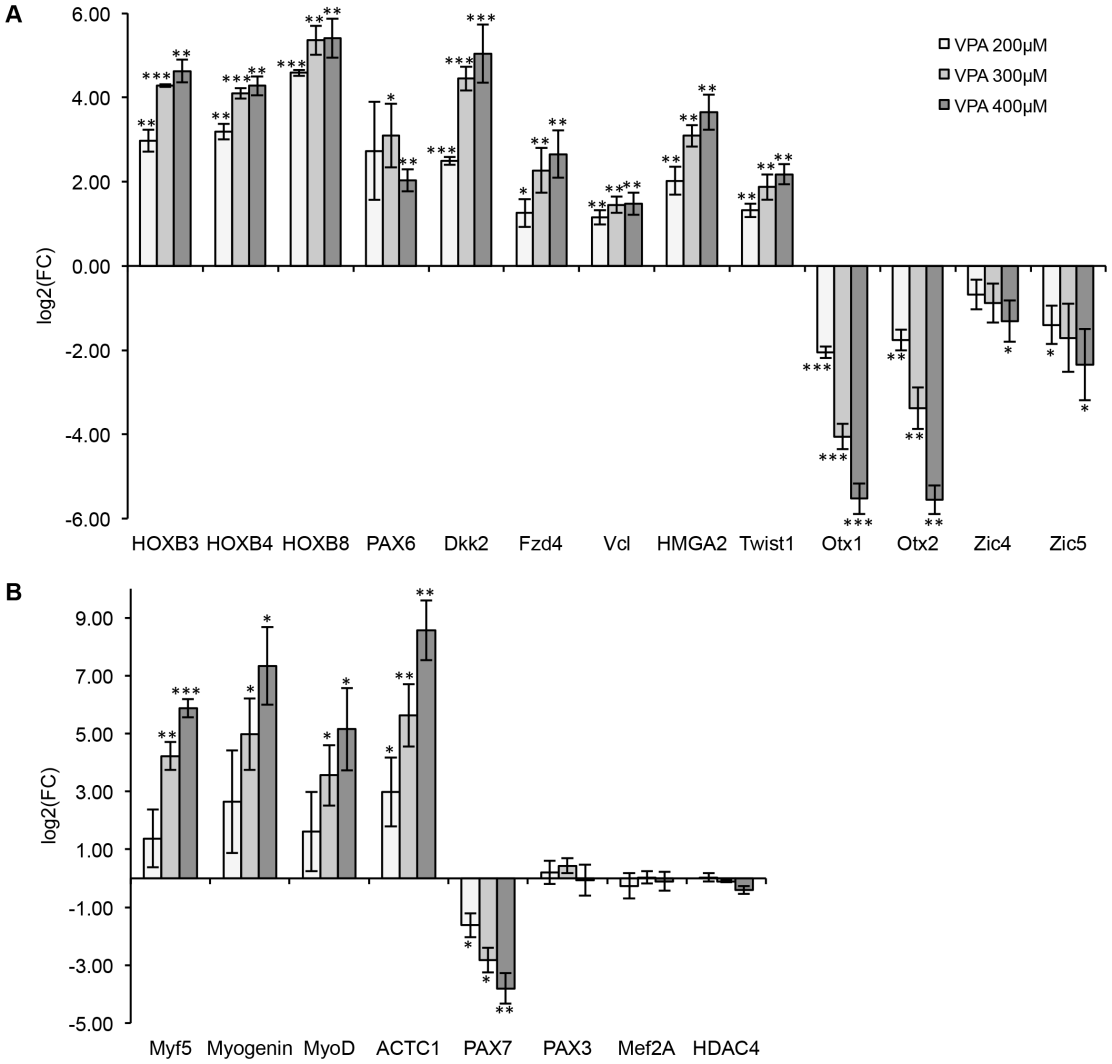
Gene expression under VPA treatment. A. RT-PCR verification of Affymetrix whole genome array data. The graph demonstrates mean of log_2_ fold change in three independent differentiation processes (VPA vs. solvent control) ± SEM for nine upregulated and four downregulated mRNAs. (n = 3 independent biological replicates, t-test, *p<0.05, **p<0.01, ***p<0.001). B. Induction of expression of myogenic regulation factors (MRFs) by VPA in neural-differentiated ES cells. The graph demonstrates mean of log_2_ fold change (VPA vs. solvent control) ± SEM. (n = 3 independent biological replicates, t-test, *p<0.05, **p<0.01, ***p<0.001).

In order to verify whole genome microarray data by real-time RT-PCR, we selected genes known to be involved in the development and neural tube closure (*Hox* genes, *Twist1, Hamga2, Pax6, Otx1, Otx2, Zic4* and *Zic5, Fzd4, Dkk2, Vcl*), as well as muscle-specific *Actc1*. We could confirm the microarray data by RT-PCR for all selected genes (Fig. 6A). The most upregulated mRNA in microarray experiments was *Actc1* that encodes α-actin, a major component of muscle tissue (Fig. 6B).

**Figure 7:**
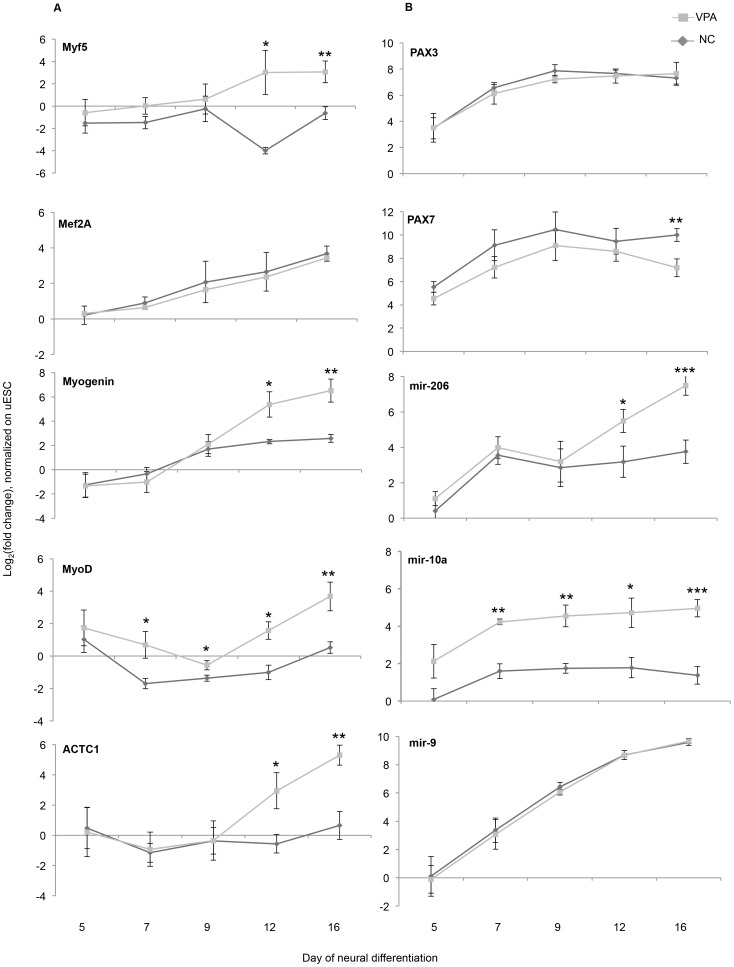
Time kinetics of MFRs and miRNA expression under VPA exposure. mES cells were treated with 300 µM VPA from day 1 of neural differentiation. RNA samples were collected on day 5, 7, 9, 12, and 16 of differentiation. The expression of genes at each time point of differentiation is normalized to the expression in undifferentiated mES cells. The graphs demonstrate mean of log_2_ fold change in three independent biological replicates (differentiated vs. undifferentiated) ± SEM. (n = 3, t-test, *p<0.05, **p<0.01, ***p<0.001).

Next we addressed the effect of VPA on several myogenic regulatory factors (MRF) known to control muscle cell differentiation, in particular *Mef2A, Myf5, MoyD, myogenin* as well as the paired box (*Pax*) transcription factors *Pax3* and *Pax7* that are known to trigger myogenesis through interplay with each other and with myo-mirs (reviewed in [54], [55]). In the first step we were able to demonstrate a concentration-dependent induction in the expression of *Myf5, myogenin*, and *MyoD*, but not *Mef2A* and *Pax3* in VPA-treated samples on day 16 of neural differentiation. *Pax7* expression was significantly reduced (Fig. 6B). The expression of *Hdac4*, a validated *mir-206* target, remained unchanged, suggesting that the regulation of this transcript by *mir-206* can probably occur at the post-transcriptional level and does not affect mRNA expression. To understand which transcription factor or miRNA expression was affected first by VPA treatment, and thus possibly contributing to the stimulation of myogenic differentiation, we analyzed the time kinetics of the expression of myo-genes together with crucial miRNAs (*mir-206, mir-10a*) and marker for differentiated muscle tissue (*Actc1*) in the course of differentiation. The expression of these genes was analyzed in undifferentiated mESCs as well as on days 5, 7 9, 12 and 16 after induction of neural differentiation under exposure to 300 µM VPA. The gene expression at each time point was normalized to the expression in undifferentiated cells (Fig. 7). The expression of three-out-of-four tested MRFs (*Myf5, MyoD, myogenin*) was affected by VPA, although their expression during differentiation was only slightly above the RT-PCR detection limit. *Myogenin* was induced up to 5 times on day 9 in both untreated and VPA-treated cells, then remained unchanged in untreated cells but was further and significantly (p<0.05) induced up to 65-fold in VPA-treated samples. *Myf5* was not induced in untreated cells and significantly increased (p<0.05) from the beginning of day 12 of differentiation in VPA-treated cells, similar to *Actc1* kinetics. *MyoD* has shown a biphasic expression profile during differentiation under VPA exposure. It was slightly induced on day 5 of differentiation (5-fold in VPA-treated samples and 2-fold in untreated cells), then downregulated on days 7 and 9, and then significantly upregulated (p<0.05) again on days 12 and 16, but only in VPA-treated samples. The induction of *MyoD* in early stages of differentiation by VPA could be explained based on the finding that proliferating myoblasts express *MyoD* [54]. The expression of *Myf2A* was induced in the course of neural differentiation beginning from day 7 in both untreated and VPA-treated samples in comparison to undifferentiated mESCs. Altogether, MRFs were significantly affected by VPA as early as day 12 of neural differentiation (Fig. 7A). Transcription factors *Pax 3* and *Pax 7*, which are known to be regulated during early stages of neural and muscle differentiation (including neural tube closure) were strongly induced in the course of neural differentiation. VPA treatment did not affect expression of *Pax 3* but significantly reduced (p<0.01) the expression of *Pax 7* as early as on day 16 of differentiation. Neither *mir-206* nor *mir-10a* could be detected in undifferentiated mESCs. As expected, we observed a significant induction (p<0.05) of *mir-206* expression in VPA-treated cells as early as on day 12 of differentiation, while *mir-10a* was strongly induced in VPA-treated but not in control samples from day 5 of differentiation, suggesting that this miRNA could be the first signal stimulating myogenesis under VPA exposure during neural differentiation of mESCs (Fig. 7B).

### Morphological confirmation of the induction of myogenesis by VPA during neural differentiation of mESCs

**Figure 8:**
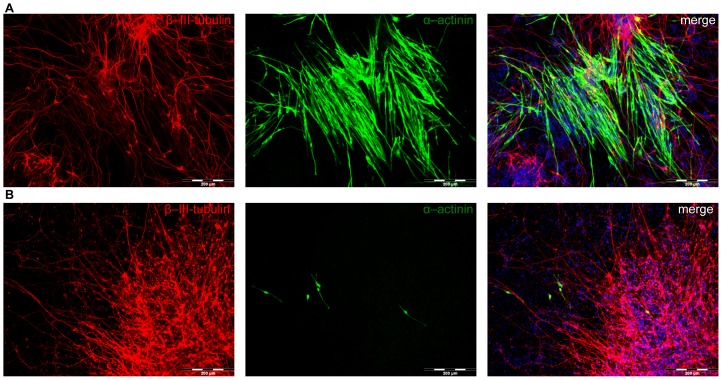
Expression of neuron- and myocyte-markers under VPA exposure. Neural-differentiated ES cells were immuno-stained with neuron specific marker β-III-tubulin (red) and muscle specific marker α-actinin (green) after VPA (A) or PBS (B) treatment.

We performed immunocytochemistry staining with an antibody against the muscle-specific marker α-sarcomeric actinin to confirm the gene expression data morphologically. We observed a 10-fold increase of α-actinin-positive cells in VPA-treated samples in comparison to the negative control (n = 3, Fig. 8, Fig. S5). Several clusters containing hundreds of α-actinin-positive cells per slide were observed in VPA treated samples, while only few sporadically distributed single α-actinin-positive cells were observed in PBS treated samples (40 cells per slide in average). No increases in α-actinin-positive cells were detected in arsenite-treated samples in comparison to solvent control (data not shown). The staining with the neuron-specific marker βIII-tubulin was performed in parallel.

### Induction of myogenic miRNAs expression during neural differentiation of mESCs is a common phenomenon for HDAC inhibitors

To examine whether the effects observed in VPA-treated samples are common for HDAC inhibitors, we induced the neural differentiation of mESCs under exposure to sub-toxic concentrations (0.75, 1 and 2 µM, Fig. S4B) of trichostatin A (TSA), a structurally unrelated HDAC inhibitor.

**Figure 9:**
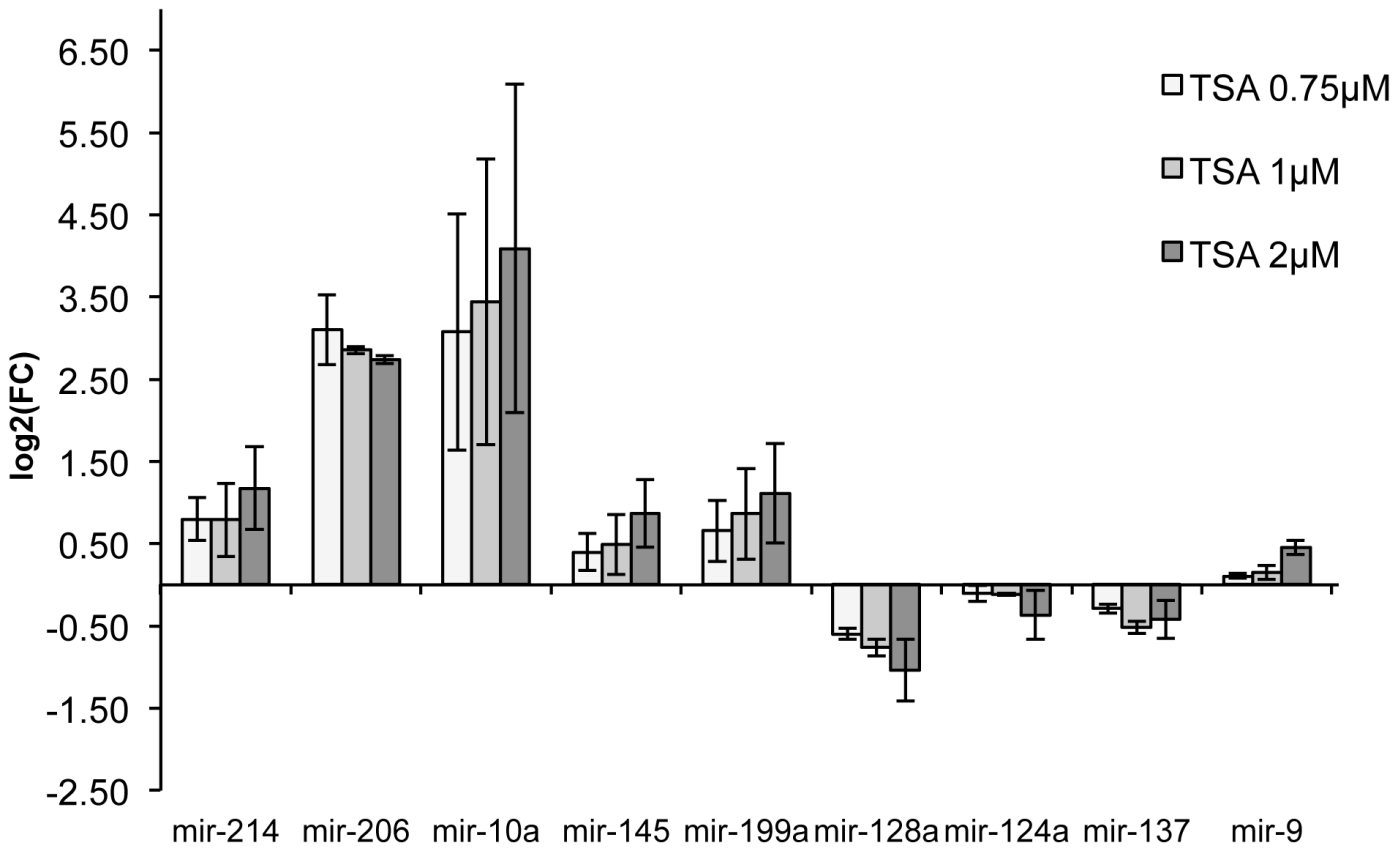
TSA induction of *mir-206* and *mir-10a* during neural differentiation of mES cells. The graph demonstrates mean of log_2_ fold change (TSA vs. solvent control) in two independent biological replicates ± SEM.

As expected, we observed the induction of *mir-206* and *mir-10a* expression upon TSA treatment. *Mir-214, mir-199a* and *mir-145* were also induced but not to such an extent as seen for VPA. *Mir-128* and *mir-137* were downregulated, while *mir-124* was not. A slight induction of *mir-9* was observed at the highest concentrations of TSA (Fig. 9). The results suggest that the induction of myogenic miRNA expression by VPA might be at least partially mediated by HDAC inhibition.

## Discussion

In this study we analyzed perturbations in the miRNome in neural-differentiated mESCs exposed to the developmental neurotoxicant VPA to establish this endpoint as a suitable tool for the prediction of developmental neurotoxicity *in vitro* and to elucidate the molecular mechanisms of VPA-dependent developmental neurotoxicity. Using microarray technology we could demonstrate substance-specific effects on miRNA expression. VPA, but not arsenite, induced the expression of muscle-specific miRNAs during neural differentiation of mESCs. By contrast, the expression of a panel of neural-specific/abundant miRNAs was found repressed by VPA (Fig. 3, 5). VPA-specific miRNA expression patterns were further confirmed at the level of mRNA by demonstrating the induction of muscle-specific genes and the accompanied inhibition of genes involved in neurogenesis (Fig. 4, 6, 7), and phenotypically by immunofluorescent staining (Fig. 8). Supporting functional analysis of VPA-specific miRNA targets performed by IPA pathway analysis revealed that VPA might affect muscle and skeletal system development, cellular and tissue development, cellular growth, proliferation, and apoptosis (Table 1). In addition, we observed similar effects on muscle-specific miRNAs with the structurally unrelated but well characterized HDAC inhibitor TSA, suggesting that the VPA effect is at least partially dependent on its known HDAC inhibitor activity (Fig. 9). In line with these observations, Gurvich and coworkers have analyzed alterations in the gene expression pattern of zebrafish and *Xenopus* embryos upon treatment with VPA or TSA and have shown that both HDAC inhibitors exert common effects at the gene expression level in these two different vertebrate model systems [56]. One of the greatest advantages of using miRNA profiling as a tool for toxicity testing is their conservation throughout the animal kingdom/the phyla. For instance, the first discovered *C. elegans* miRNAs, *let-7* and *lin-4* (*mir-125* for mammals), their targets and functions, have been proven to be conserved in regulating developmental timing, stem cell differentiation and neural development in worms, flies and mammals [57]–[60]. Most regulated miRNAs shown in our study are highly conserved between mice and humans (e.g. *mir-206, mir-214, mir-10a, mir-124, mir-137, mir-128, mir-9*) [61], [62]. Myogenesis regulating *mir-206* is highly expressed in skeletal muscles in both species [62], [63] as is *mir-124, mir-9, mir-128 and mir-137* in mouse and human brain where they are responsible for fine-tuning of neurogenesis [62] [64]. Importantly, clustering of some miRNA genes within the genome is also conserved between human and mice (e.g. *mir-206/mir-133b*, *mir-214/mir199a*, *mir-10a/HoxB4*, *mir-145/143* clusters, all of which have been studied here) (www.miRbase.org [61]). Hence, miRNA profiling may help to overcome interspecies differences what is certainly one of the obstacles in using non-human cell lines to predict human toxicity. Nevertheless, similar experiments using human embryonic stem cells or induced pluripotent stem cells (iPSC) will answer the question whether the effects will be applicable for human cells and human neural development in general.

The *mir-206*, a skeletal muscle-specific miRNA, was the most strongly upregulated miRNA in our experimental system (up to 100-fold after exposure, Fig. 3, 5 and Table S2). Expression of *mir-206* was also found strongly induced during differentiation of C2C12 myoblasts [65], [66]. This miRNA promotes skeletal myogenesis by translational but not transcriptional repression of multiple targets including *Hdac4*, a key regulator of myogenesis [66]. In agreement with these observations, we did not observe any VPA-induced changes in *Hdac4* transcription levels during the differentiation of mESCs. In addition, *mir-206* regulates the developmental timing of muscle differentiation by targeting early markers of myogenesis, such as *Pax3* and *Pax7* [67], [68]. *Pax7* is highly expressed in proliferating myoblasts, where it represses the expression of myogenic factors like *MyoD*. Thus, *mir-206* can stimulate the expression of *MyoD* by targeting and translational repression of *Pax7*. On the other hand, *mir-206* itself is transcriptionally regulated by *MyoD*, meaning that both molecules reciprocally regulate each other while driving myogenesis (reviewed in [55]). These changes in the differentiation process of muscle tissue also correlate with the observed transcriptional downregulation of *Pax7* and the transcriptional induction of *MyoD* by VPA (Fig. 6B). Interestingly, *mir-206* is also known as key regulator of the signaling between motor neurons and skeletal muscles at neuromuscular junctions and is required for proper formation and/or regeneration of neuromuscular synapses after injury by targeting *Hdac4* and *Connexin43* [69], [70].

*Mir-10a* and *10b* are both highly evolutionarily conserved and localized within *Hox* gene clusters. *Hox* genes are conserved transcription factors, regulating the anterior-posterior pattern formation during development and have been implicated in neural tube closure [71]. *Mir-10a/b* as well as a number of *Hox* genes (Table S4) were strongly induced during neural differentiation under VPA exposure in our mESC system (23.9/4.3-fold, Fig. 3, 5 and Table S2). Both miRNAs are upregulated in response to retinoic acid, a common inducer of cellular differentiation in different cell types and *mir-10a/b* were shown to contribute to retinoic acid-induced differentiation of neuroblastoma cells [72], [73]. Huang and co-authors were able to demonstrate that *mir-10a* expression is strongly increased upon retinoic acid-induced muscle differentiation of ESCs, where it determines the smooth muscle lineage by targeting *Hdac4* [72]. The finding that *mir-10a* is the earliest upregulated gene, which, in contrast to myogenic factors or *mir-206*, already displayed a significant increase in the expression level on day 5 of differentiation in VPA-treated mESCs, suggests that it plays a central role in the stimulation of myogenesis during VPA-mediated disturbance of neural differentiation. In addition, only *mir-10a* was found upregulated after VPA treatment of primary cortical neurons (Fig. S3), thus suggesting that the expression of *mir-10a* might be directly affected by VPA even at later stages of neural development. On the contrary, *mir-206* was not upregulated in primary cultures, suggesting that the strong upregulation observed during neural differentiation of mESCs is (i) a result of myogenic lineage specification triggered by exposure to VPA at earlier stages of differentiation process, and (ii) that VPA is unable to directly stimulate the expression of *mir-206* in already committed cells. For the same reason we did not observe any differences in the expression of neuron-specific miRNA in primary cultures (*mir-124, mir-128*, Fig. S3), which are known to play a significant role in earlier, but not later stages, of neurogenesis. In addition, the fact that about 40% of all known miRNAs could be affected by HDAC inhibitors while the percentage of protein encoding genes rather remains low [38] reinforces our hypothesis that *mir-10a* could be a first and direct target of VPA. In the future studies chromatin immunoprecipitation (CHIP) analysis of the *mir-10* promoter region may be helpful to clarify whether VPA induces the acetylation/activation of histone complexes in the regulatory regions of the *mir-10a* locus.

We also observed a VPA-dependent induction of myogenic regulatory factors (*MyoD, Myf5, and myogenin*, Fig. 6 and 7) as well as an increased number of α-actinin-positive myocytes in neural-differentiated mESCs (Fig. 8). These results further support the findings on the miRNA level. It was previously shown that HDAC inhibitors, including VPA, enhance differentiation of myoblasts by regulation of MRFs [74], [75]. This, together with the observation that ectopic expression of myogenic factors (such as *Pax3, MoyD, Myf5* and *myogenin*) in neural tubes of chicken embryos activates muscle-specific gene expression and myogenesis in neural tissue [76] might explain the upregulation of myo-mirs and MRFs in our system and, as consequence, an activation of myogenesis by VPA during neural differentiation of mESCs.

Mir-214/mir-214* and *mir-199a-5p/mir199a-3p* are clustered *on* pri*-mir-199a-2* within the *Dnm3os* gene in mouse as well as in human genome and were strongly upregulated by VPA (8.7/20.6- and 9.8/11.1-fold, respectively), while exposure to arsenite significantly reduced the expression of these miRNAs (−2.4/−1.97- and −2.4/−2.9-fold, respectively). *The mir-214/mir-199a* cluster is regulated during embryonic development by transcription factor Twist1 [77], [78], the expression of which was significantly induced by VPA in our cell system (2.2-fold). Twist1 is implicated in the differentiation of multiple cell lineages including muscle formation, and regulates maturation and differentiation of human ovarian cancer stem cells through induction of the aforementioned miRNAs and subsequent inhibition of *Pten* and *Ikkβ* by those miRNAs that lead to inhibition of NF-κB and activation of Akt pathways [77]. In addition, Twist1 plays a significant role in mesoderm formation and epithelial mesenchymal transition, which is a hallmark of gastrulation, and particularly in neural crest specification and migration [79], [80]. *Mir-214* was shown to specify muscle cell fate in the embryonic development of zebrafish by modulation of Hedgehog signaling during early segmentation stages [81]. *Mir-214* is expressed in C2C12 myoblasts and strongly induced in the course of differentiation where it regulates both proliferation and differentiation depending on the culturing conditions, probably by targeting negative regulators of MRFs [82]. Furthermore, the histone methyltransferase Ezh2, a polycomb group protein, as well as N-Ras, both of which known repressors of skeletal myogenesis, were shown to be negatively regulated by *mir-214* during muscle differentiation [83], [84]. *In situ* hybridization of regulated miRNA may contribute to the understanding of the role of these miRNA in differentiated cells and clarify whether the expression of *mir-214* is restricted to muscle cells or induced in neural cells as well.

VPA treatment of mESCs during neural differentiation led to the downregulation of some crucial neuronal miRNAs. The most VPA-sensitive miRNA was *mir-137* (3.8-fold downregulated in comparison to control). *Mir-137* is required for neural differentiation of ESCs [85] and modulates differentiation of adult mouse neural stem cells as well as brain tumor stem cells by crosstalk with methylation agents [86], [87].

*Mir-9* and *mir-124*, the two most abundant miRNAs in the CNS, showed different responses to VPA treatment: while *mir-124* was downregulated, no effect on *mir-9* expression could be observed after VPA exposure. Both were shown to promote neuronal lineage commitment by targeting multiple anti-neuronal factors in neuronal cells (reviewed in [16]). These results suggest that VPA affects neural differentiation processes and pathways, which are specific for *mir-124* but not *mir-9*. Exposure of differentiating mESCs to VPA also reduced expression of *mir-128*, which is an enhancer of neural differentiation [88]. Interestingly, a negative regulator of dendrite outgrowth and maintenance, *mir-375* [89], was significantly upregulated (2-fold) by VPA in neural-differentiated mESCs.

The effect of VPA on transcriptome during neural differentiation was extensively studied in hESC ([90]–[93]), mESC systems [24], [94] and P19 mouse embryonic carcinoma cells ([51], [52]). However, these studies did not address the question whether VPA may perturb miRNA expression during neural differentiation. In agreement with aforementioned studies we observed perturbations in expression of the genes associated with neural tube defects, HDAC inhibition pathways, genes involved in embryonic development especially neural development. Neither of these studies reported induction of myogenesis or lineage shift that can be explained by differences in exposure time: our cell system was exposed throughout the whole differentiation process, beginning from the first day of differentiation, while Theunissen et al. and Jergil et al. performed transcriptomics analysis after 24 hours of exposure (at day 11 of differentiation or in undifferentiated embryonic stem cells, respectively). Leist and colleagues analyzed perturbations in gene expression by VPA at earlier stages of differentiation (stage of neural ectodermal progenitor cells, four and six days after induction of differentiation). Overall, performing short-term exposure allows for identification of genes involved in the initial cellular response to the toxicant, while changes in the differentiation process such as lineage shifts rather will be measured upon long-term exposures covering the entire differentiation process.

## Conclusions

The findings presented here may contribute to a better understanding of neural tube defects induced by VPA. As the data shows, one of the main advantages of miRNome against whole transcriptome profiling is the amount of data: there are 2578 mature human and 1908 mouse miRNAs identified so far (http://www.mirbase.org) [95]). Therefore, miRNA profiling provides a more compact data set that might facilitate the analysis of a specific substance's effects to greater extent. In addition, miRNA profiling may have stronger predictively than whole genome profiling, as miRNAs were shown to be crucial development regulators, influencing developmental timing as well as cell specification (reviewed in [15]). An explosive number of studies analyzing miRNA functions in numerous cellular processes, especially neural development, open a new dimension for analysis of pathways of toxicity, where miRNAs might be involved. Thus, miRNA profiling may serve as an accomplishment to the transcriptomics and as a good molecular marker for developmental neurotoxicity testing and for discovery of new pathways of toxicity. Based on miRNA profiling we could demonstrate an unexpected and previously unknown adverse activity of VPA on neural differentiation of mESCs inducing a shift in lineage determination from neurogenesis into myogenesis. The mechanisms underlying these observations are unclear. However, it is tempting to speculate that some of the aforementioned miRNAs could be primary targets of HDAC inhibition that at least partially may trigger myogenesis in our system. In general, analysis of miRNA expression profiles after VPA exposure provided the first glance of certain miRNAs targets that might be involved in mediating the developmental neurotoxicity of this substance.

## Supporting Information

Figure S1 **Monitoring of stemness and neural differentiation of mES cells.** A. Undifferentiated ES cells line W4 expressed alkaline phosphatase, Oct3/4 and Nanog (96% Oct3/4- and 77% Nanog-positive cells in the population measured by flow cytometry). B. Western blot stained with antibodies against ES cells marker (Oct4), neuronal marker (β-III-Tubulin), glial marker (GFAP) and β-actin as a loading control in undifferentiated ES cells as well on day 12 and 21 of differentiation. C. Flow cytometry quantification of neuronal marker expression, MAP2 and β-III-Tubulin, on day 12 of differentiation. The flow cytometry plots depict the PE positive cells in percent (y-axis) vs. Side Scatter. Gates (x-axis) were gated based on negative controls lacking first antibody. D. The neuronal morphology was visualized on day 9, 12, and 16 of neural differentiation by immunostaining with Nestin, MAP2 and β-III Tubulin. Presence of astroglia cells was demonstrated by the staining with antibody against GFAP. E. Expression of neural specific or enriched miRNAs was strongly induced on day 16 of neural differentiation. The graph represents mean of absolute miRNA expression levels normalized to undifferentiated ES cells measured in four independent RT-PCR experiments. β-III-Tubulin expression was analyzed in parallel as a positive control of neural differentiation. F. Expression of neuronal specific markers *mir-9*, *mir-124* and *β-III-tubulin* and neuro-progenitor specific marker *nestin* at different time points of neural differentiation. The expression of genes at each time point of differentiation is normalized to the expression in undifferentiated mES cells. The graph demonstrates mean of log_2_ fold change in three independent biological replicates (differentiated vs. undifferentiated) ± SEM.
(TIF)

Figure S2 **Molecular networks of VPA-sensitive miRNA and their reciprocal expressed target mRNA.** The IPA–generated network is a graphical representation of the molecular relationships between molecules. Molecules are represented as nodes, and the biological relationship between two nodes is represented as an edge (line). All edges are supported by at least one reference from the literature or from canonical information stored in the Ingenuity Knowledge Base. The intensity of the node color indicates the degree of up- (red) or down- (green) regulation. Nodes are displayed using various shapes that represent the functional class of the gene product.
(TIF)

Figure S3 **miRNA expression in mouse primary cortical neurons exposed to VPA for 10 days** ***in vitro*** **.** No changes could be observed in expression of neural specific miRNAs between VPA and solvent control. *mir-206* was not detected in primary cultures, while *mir-10a* was strongly induced upon VPA treatment. The graph demonstrates mean of log_2_ fold change (VPA vs. solvent control) in two independent biological replicates ± SEM.
(TIF)

Figure S4 **Toxicant (VPA, arsenite and TSA) effects on cell viability.** A. and B. The W4 ES cells were induced to differentiate into neurons for 16 days under continuous substance (arsenite (A) and TSA (B)) exposure. C. Primary neurons were exposed to VPA from day 1 until day 10 *in vitro*. Cell viability was estimated using CellTiterBlue assay and is shown as a percentage of solvent control.
(TIF)

Figure S5 **Expression of neuron- and myocyte-markers under VPA exposure: Supportive lower magnification overview images for** **Fig. 8** **.** Neural-differentiated ES cells were immuno-stained with neuron specific marker β-III-tubulin (red) and muscle specific marker α-actinin (green) after VPA (A) or PBS (B) treatment. At least five big muscle clusters (containing several hundreds of α-actinin-positive cells per slide) could be found in samples treated with VPA, while only signal sporadically distributed cells (around 40 cells in average per slide) were positive for muscle marker in PBS control.
(TIF)

Table S1 **Primers used for qRT-PCR.**
(DOCX)

Table S2 **miRNAs responding to valproate (left panel) or arsenite (right panel) treatment during neural differentiation of ESCs.** log_2_ of the mean fold change for each miRNA normalized to solvent control is given. A total of 110 miRNA were identified differently expressed in neurally differentiating mES cells under VPA treatment (300 µM) compared to solvent control on day 16 of differentiation. Exposure of neurally differentiating mES cells to arsenite (0.75 µM) altered the expression of 27 miRNAs. Threshold was set as over 2-fold change and p<0.05. Mmu – mouse musculus. Hsa- homo sapiens, hp – hairpin, pri – primary.
(DOCX)

Table S3 **Valproate and arsenite intersected miRNA.**
(DOCX)

Table S4 **Genes, responding to valproate treatment during neural differentiation of ESCs.** Log_2_ of the mean fold change for each mRNA normalized to untreated control is given. A total of 377 mRNA were identified differently expressed in neurally differentiating mES cells under VPA treatment (300 µM) compared to untreated control during 16 days of neural differentiation. Threshold was set as over 2-fold change and p<0.05.
(DOCX)

Table S5 **miRNAs and their target mRNAs, responding to valproate treatment in a reciprocal manner during neural differentiation of ESCs.** log_2_ of the mean fold change for each miRNA and mRNA normalized to untreated control is given.
(DOCX)
