# Supplementary figures and images for: MicroRNA Profiling as Tool for In Vitro Developmental Neurotoxicity Testing: The Case of Sodium Valproate
Source: PLoS One. 2014 Jun 4;9(6):e98892. doi: 10.1371/journal.pone.0098892 (PMC4045889; doi:10.1371/journal.pone.0098892)

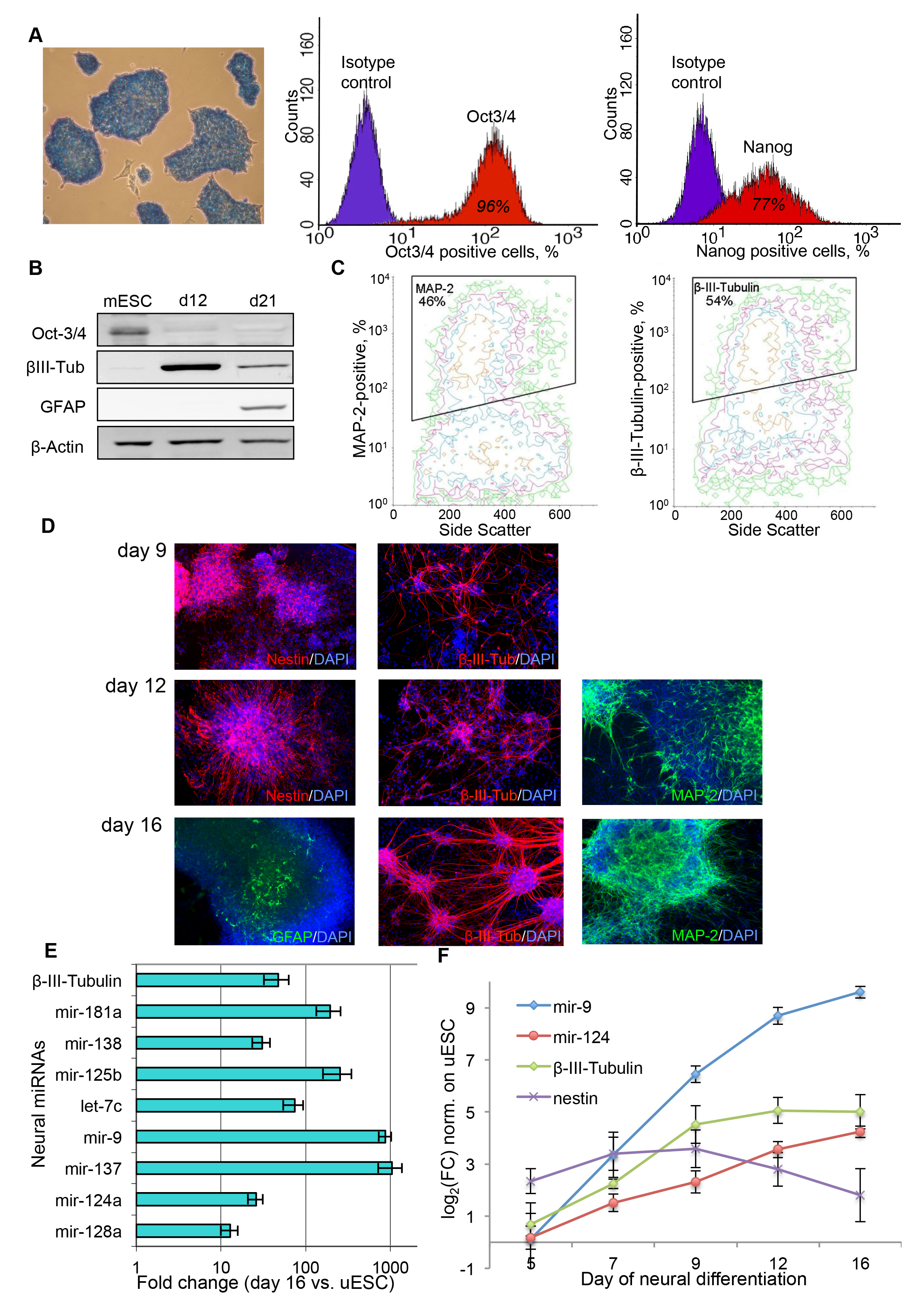

Supplement: Figure S1 — Monitoring of stemness and neural differentiation of mES cells. A. Undifferentiated ES cells line W4 expressed alkaline phosphatase, Oct3/4 and Nanog (96% Oct3/4- and 77% Nanog-positive cells in the population measured by flow cytometry). B. Western blot stained with antibodies against ES cells marker (Oct4), neuronal marker (β-III-Tubulin), glial marker (GFAP) and β-actin as a loading control in undifferentiated ES cells as well on day 12 and 21 of differentiation. C. Flow cytometry quantification of neuronal marker expression, MAP2 and β-III-Tubulin, on day 12 of differentiation. The flow cytometry plots depict the PE positive cells in percent (y-axis) vs. Side Scatter. Gates (x-axis) were gated based on negative controls lacking first antibody. D. The neuronal morphology was visualized on day 9, 12, and 16 of neural differentiation by immunostaining with Nestin, MAP2 and β-III Tubulin. Presence of astroglia cells was demonstrated by the staining with antibody against GFAP. E. Expression of neural specific or enriched miRNAs was strongly induced on day 16 of neural differentiation. The graph represents mean of absolute miRNA expression levels normalized to undifferentiated ES cells measured in four independent RT-PCR experiments. β-III-Tubulin expression was analyzed in parallel as a positive control of neural differentiation. F. Expression of neuronal specific markers mir-9, mir-124 and β-III-tubulin and neuro-progenitor specific marker nestin at different time points of neural differentiation. The expression of genes at each time point of differentiation is normalized to the expression in undifferentiated mES cells. The graph demonstrates mean of log2 fold change in three independent biological replicates (differentiated vs. undifferentiated) ± SEM. (TIF) [file pone.0098892.s001.tif]

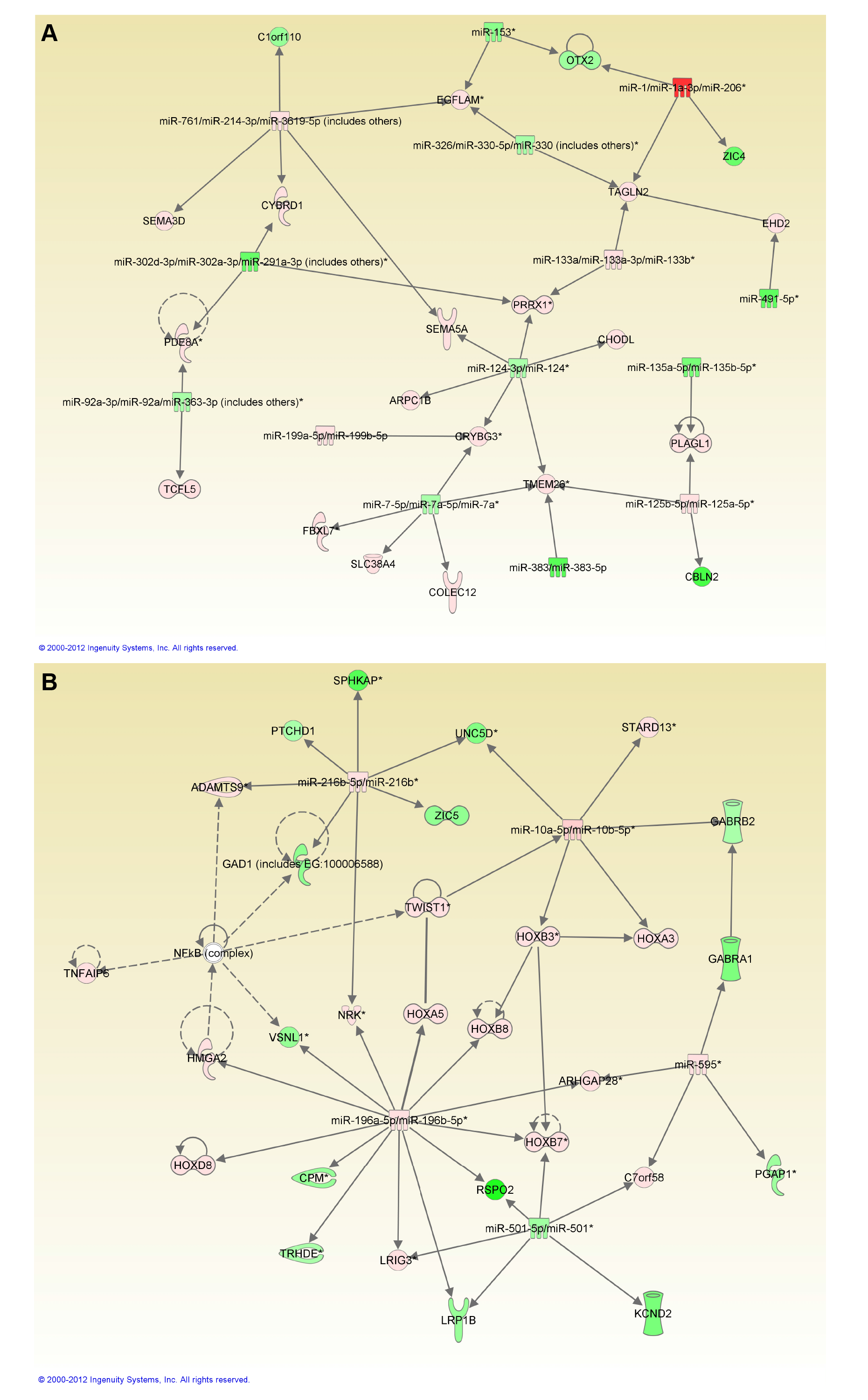

Supplement: Figure S2 — Molecular networks of VPA-sensitive miRNA and their reciprocal expressed target mRNA. The IPA–generated network is a graphical representation of the molecular relationships between molecules. Molecules are represented as nodes, and the biological relationship between two nodes is represented as an edge (line). All edges are supported by at least one reference from the literature or from canonical information stored in the Ingenuity Knowledge Base. The intensity of the node color indicates the degree of up- (red) or down- (green) regulation. Nodes are displayed using various shapes that represent the functional class of the gene product. (TIF) [file pone.0098892.s002.tif]

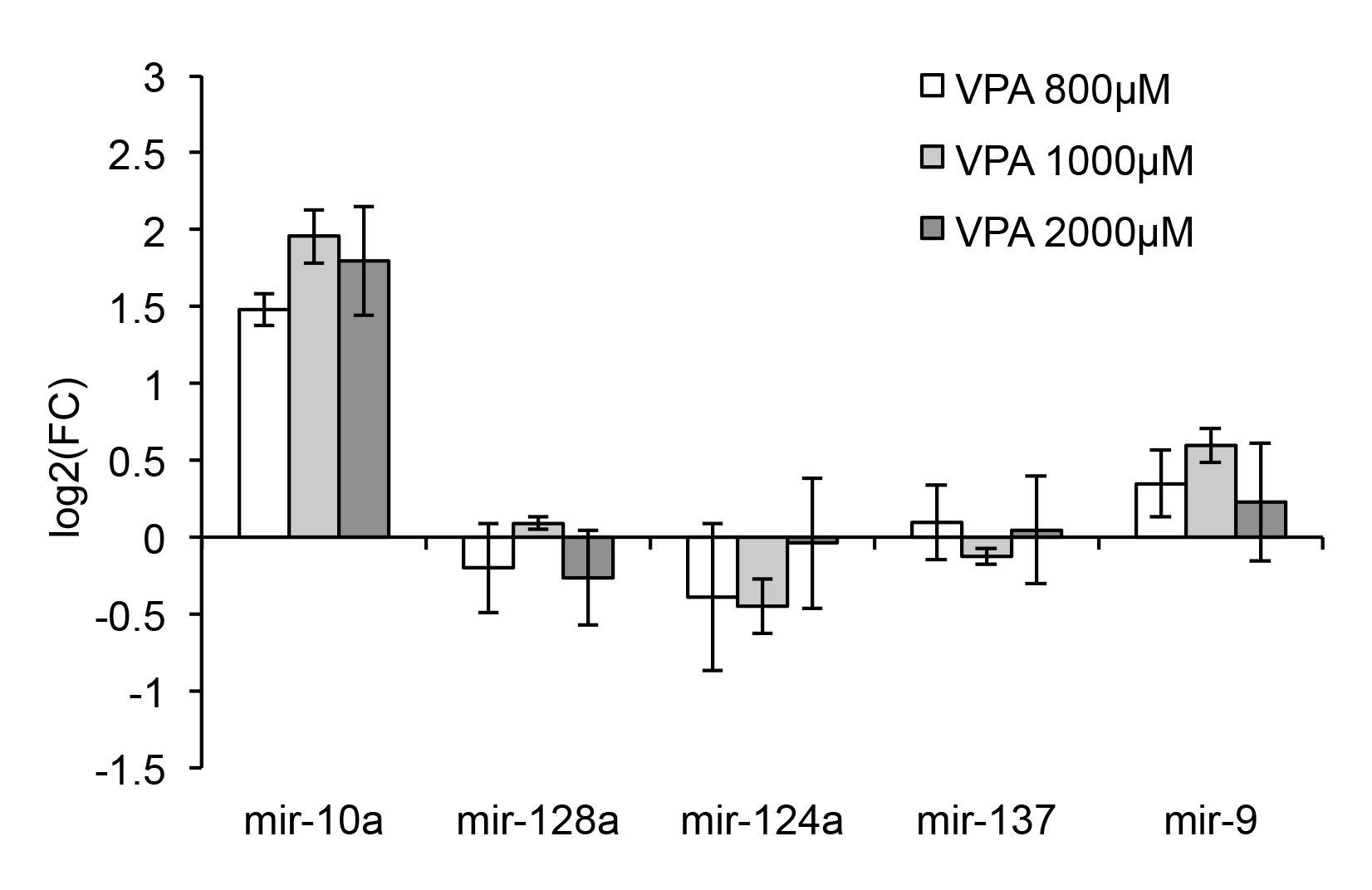

Supplement: Figure S3 — miRNA expression in mouse primary cortical neurons exposed to VPA for 10 days in vitro . No changes could be observed in expression of neural specific miRNAs between VPA and solvent control. mir-206 was not detected in primary cultures, while mir-10a was strongly induced upon VPA treatment. The graph demonstrates mean of log2 fold change (VPA vs. solvent control) in two independent biological replicates ± SEM. (TIF) [file pone.0098892.s003.tif]

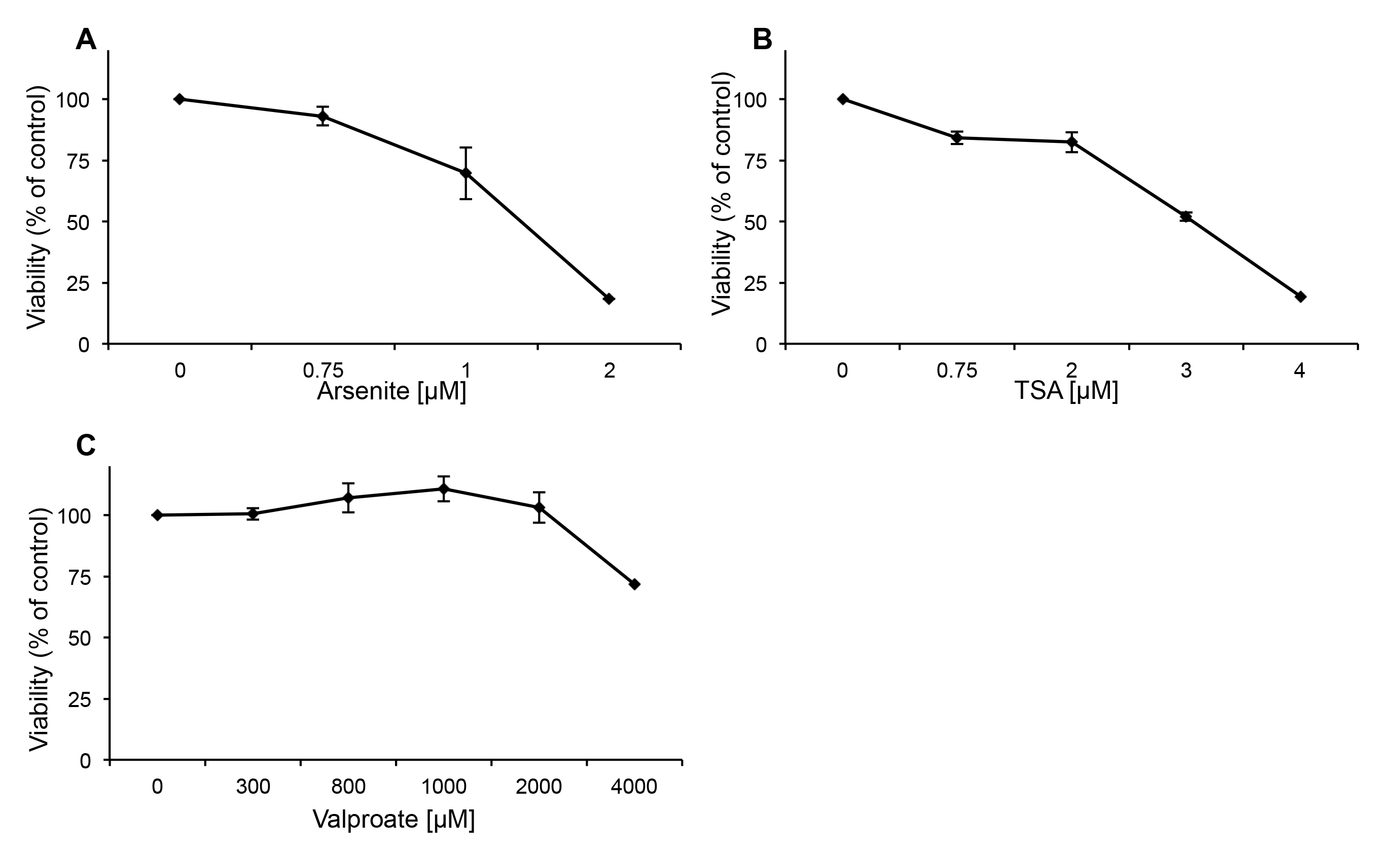

Supplement: Figure S4 — Toxicant (VPA, arsenite and TSA) effects on cell viability. A. and B. The W4 ES cells were induced to differentiate into neurons for 16 days under continuous substance (arsenite (A) and TSA (B)) exposure. C. Primary neurons were exposed to VPA from day 1 until day 10 in vitro. Cell viability was estimated using CellTiterBlue assay and is shown as a percentage of solvent control. (TIF) [file pone.0098892.s004.tif]

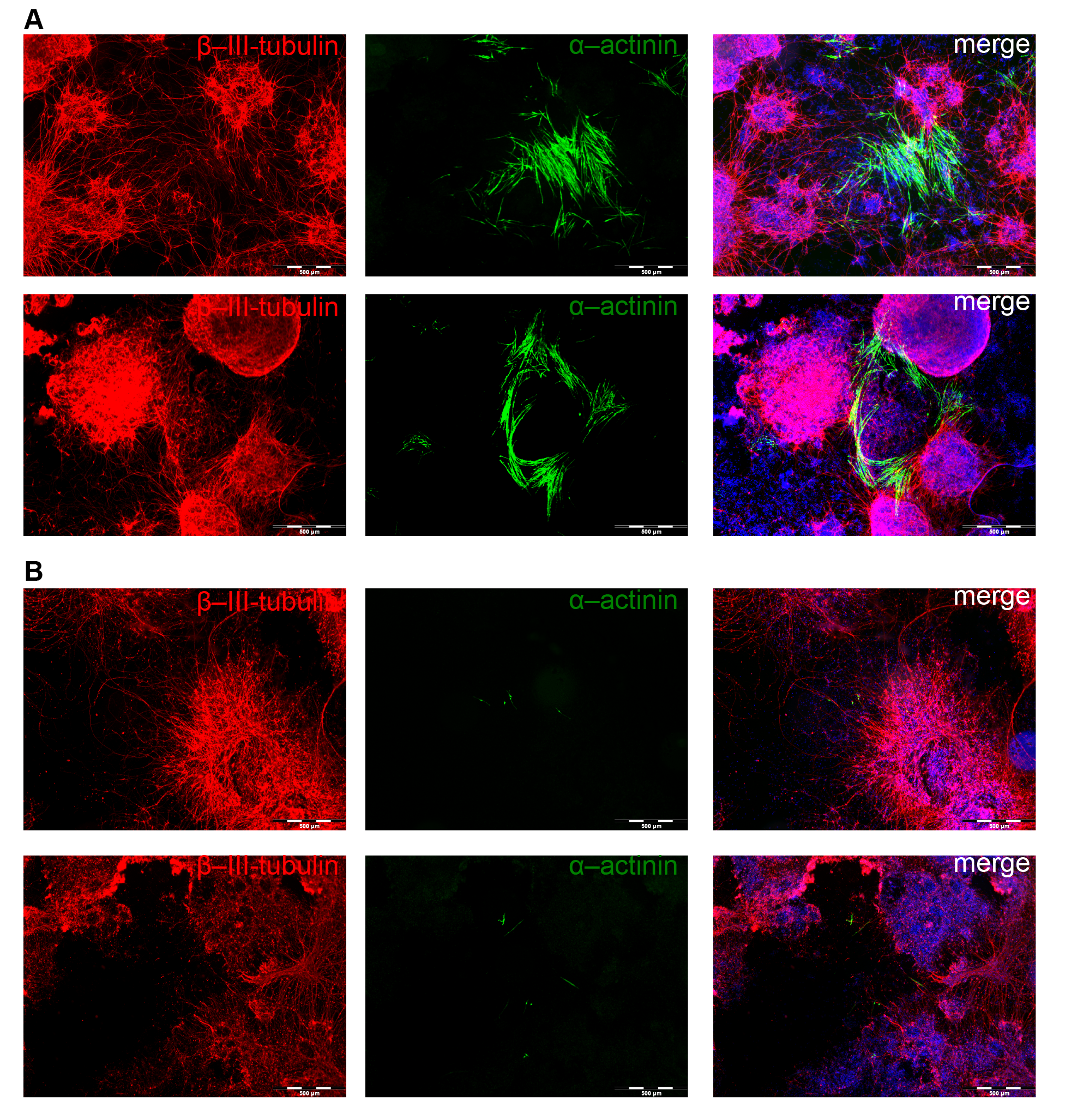

Supplement: Figure S5 — Expression of neuron- and myocyte-markers under VPA exposure: Supportive lower magnification overview images for Fig. 8 . Neural-differentiated ES cells were immuno-stained with neuron specific marker β-III-tubulin (red) and muscle specific marker α-actinin (green) after VPA (A) or PBS (B) treatment. At least five big muscle clusters (containing several hundreds of α-actinin-positive cells per slide) could be found in samples treated with VPA, while only signal sporadically distributed cells (around 40 cells in average per slide) were positive for muscle marker in PBS control. (TIF) [file pone.0098892.s005.tif]
