# Supplementary material for: MicroRNA Profiling as Tool for In Vitro Developmental Neurotoxicity Testing: The Case of Sodium Valproate
Source: PLoS One. 2014 Jun 4;9(6):e98892. doi: 10.1371/journal.pone.0098892 (PMC4045889; doi:10.1371/journal.pone.0098892)
Supplement: Table S1 — Primers used for qRT-PCR. (DOCX) [file pone.0098892.s006.docx]

Table S1. Primer used for qRT-PCR

| **Gene Name** | **Primer, Forward, 5’-3’** | **Primer, Reverse, 5’-3’** |
| --- | --- | --- |
| 18S | TTGACGGAAGGGCACCACCAG | 5’-GCACCACCACCCACGGAATCG |
| Actc1 | tggattttgagaacgagatgg | gcggatatcgatgtcacactt |
| β-III-Tubulin | ACTGCGGAGGAGGAGGGGGA | CTTCACTTGGGCCCCTGGGCT |
| Branchyury | atgctgcagtcccatgataac | tgcgtcagtggtgtgtaatgt |
| Dkk2 | tggaagatactgccacagtcc | accatggttgcgatctctatg |
| Fzd4 | gctacaacgtgaccaagatgc | ctctcaggactggttcacagc |
| HADAC4 | gcacagaggtgaagatgaagc | gtcatctttggcgtcgtacat |
| Hmga2 | caaaggcagcaaaaacaagag | ttgcgaggatgtctcttcagt |
| HoxB3 | TGAACGGGACCAGAGGAGCTCAG | AGCCACCGACGAGGGGAGAA |
| HoxB4 | GCGTGCAAAGAGCCCGTCGT | CCGGCGTAATTGGGGTTTACCGTG |
| HoxB8 | CTTTCCCTGGATGCGCCCTCA | CGCGTGCGATACCTCGATCCTCC |
| Mef2A | CGGCAGCCCTGGACTAGAAGGT | CGCCCCATTTTCAGTCACCTTGTCT |
| Myf5 | GCTCTGACGGCATGCCTGAATGT | AGCAATCCAAGCTGGACACGGA |
| MyoD | TGGCATGATGGATTACAGCGGCCC | GAGGCTCGACACAGCCGCAC |
| Myogenin | CAACTCCCACAGCGCCTCCTG | CGTCTGTAGGGTCAGCCGCGA |
| Nestin | AGGCTGAGAACTCTCGCTTGC | GGTGCTGGTCCTCTGGTATCC |
| Otx1 | AAGCCACTCCGACAAGGTTGGCT | CAGTTCAGGGCTCCCACTGCG |
| Otx2 | ACTTGCCAGAATCCAGGGTGCAG | AGCTCTTCTTCTTGGCAGGCCTCA |
| Pax3 | CCTCCATCGGGGCCCTCCAA | GGCCCCCAGTGAGAGGGGAG |
| PAX6 | ACCTGACACCCCACCCTCGG | ACGGGGCTCTGAGAACTGGGA |
| Pax7 | GCTTGCCCACGTCCCAGTCT | CGCTGTGTGGACAGGCTCACG |
| pri-mir10a | TCCTGGGCCAAGAAGACCTGT | AGGCAGTCACTGGATTGTCCTCA |
| pri-mir-124-1 | ATGGGGCTGTCTGAGCACCTTG | CTACCCGTACTGTGGGCGCCT |
| pri-mir-124-2 | GGCTGCACTTGAAGGACATCCGA | ATGCGAGGGGTCCTTGTGTCG |
| pri-mir-124-3 | CCTCCGCCGCTCCTTTCTCATG | GATTTGTCCGCGGAGCGAGG |
| pri-mir-128-1 | GCCCTGACTCTTTGATACGTAGCTGC | CAGCTCAGGAAGAAGGCCAATTATTG |
| pri-mir-206 | TGCTCCCCATCCTAGCCCCTT | ACTAGGACCCCGGGAGCTGA |
| pri-mir9 | GGCAGCCTTGTGAGGGAAGCG | CGTTCCTCGGTGACCTTGAAGGAG |
| Twist | gagcaagattcagaccctcaa | tccttctctggaaacaatgaca |
| Vcl | GCCAGGCAGCTCCACGATGAA | TGCCATCAGCAGAGCCATGCG |
| Zic4 | GGTGATCCGGGCAACAGGTGG | ACCGCTTCCTCATCACCAGGGA |
| Zic5 | cgagaacctcaagatccacaa | cttgggtgggtgtacgattta |
