# Supplementary material for: MicroRNA Profiling as Tool for In Vitro Developmental Neurotoxicity Testing: The Case of Sodium Valproate
Source: PLoS One. 2014 Jun 4;9(6):e98892. doi: 10.1371/journal.pone.0098892 (PMC4045889; doi:10.1371/journal.pone.0098892)
Supplement: Table S2 — miRNAs responding to valproate (left panel) or arsenite (right panel) treatment during neural differentiation of ESCs. log2 of the mean fold change for each miRNA normalized to solvent control is given. A total of 110 miRNA were identified differently expressed in neurally differentiating mES cells under VPA treatment (300 µM) compared to solvent control on day 16 of differentiation. Exposure of neurally differentiating mES cells to arsenite (0.75 µM) altered the expression of 27 miRNAs. Threshold was set as over 2-fold change and p<0.05. Mmu – mouse musculus. Hsa- homo sapiens, hp – hairpin, pri – primary. (DOCX) [file pone.0098892.s007.docx]

Table S2: miRNAs responding to valproate (left panel) or arsenite (right panel) treatment during neural differentiation of ESCs.

| **Valproate** | | |  | **Arsenite** | | |
| --- | --- | --- | --- | --- | --- | --- |
| **miRNA Name** | **log_2_(FC)** | **p-Value** |  | **miRNA Name** | **log_2_(FC)** | **p-Value** |
| mmu-miR-383 | **-2.05** | 1.25E-06 |  | mmu-miR-199a-3p | **-1.54** | 6.63E-03 |
| hsa-miR-383 | **-2.01** | 1.09E-06 |  | hsa-miR-2278 | **-1.44** | 1.13E-02 |
| mmu-miR-491 | **-1.98** | 6.67E-06 |  | mmu-miR-199b | **-1.43** | 2.34E-04 |
| mmu-miR-137 | **-1.93** | 9.09E-05 |  | hp_mmu-mir-466f-4x | **-1.30** | 1.63E-02 |
| mmu-miR-302d | **-1.91** | 6.42E-05 |  | mmu-miR-199a-5p | **-1.25** | 8.30E-03 |
| hsa-miR-1251 | **-1.85** | 4.62E-07 |  | mmu-miR-214 | **-1.24** | 2.19E-04 |
| mmu-miR-129-3p | **-1.81** | 1.74E-05 |  | hp_hsa-mir-9-1 | **-1.19** | 2.78E-02 |
| pri-mmu_mir135a | **-1.75** | 2.89E-05 |  | hsa-miR-448 | **-1.18** | 3.54E-04 |
| mmu-miR-449a | **-1.72** | 9.82E-04 |  | hsa-miR-383 | **-1.09** | 5.75E-04 |
| hsa-miR-486-3p | **-1.65** | 9.88E-05 |  | mmu-miR-325 | **-1.06** | 3.89E-02 |
| pri-mmu-mir384 | **-1.60** | 1.03E-03 |  | mmu-miR-145 | **-1.02** | 2.38E-02 |
| mmu-miR-135a* | **-1.54** | 3.05E-05 |  | mmu-miR-383 | **-1.01** | 1.38E-03 |
| mmu-miR-7a* | **-1.51** | 1.04E-02 |  | hsa-miR-182 | **1.01** | 1.83E-05 |
| mmu-miR-153 | **-1.51** | 9.88E-07 |  | mmu-miR-24-2* | **1.08** | 1.93E-03 |
| mmu-miR-129-5p | **-1.48** | 2.63E-07 |  | mmu-miR-497 | **1.09** | 3.17E-02 |
| hsa-miR-181c* | **-1.43** | 1.04E-06 |  | mmu-miR-204 | **1.11** | 4.01E-03 |
| hsa-miR-4324 | **-1.29** | 2.86E-03 |  | mmu-miR-331-5p | **1.14** | 2.49E-02 |
| hsa-miR-135b* | **-1.26** | 9.09E-03 |  | mmu-miR-203 | **1.14** | 3.29E-02 |
| mmu-miR-210 | **-1.25** | 2.16E-06 |  | hsa-miR-34a* | **1.17** | 2.87E-03 |
| mmu-miR-501-5p | **-1.22** | 4.50E-02 |  | mmu-miR-205 | **1.17** | 5.58E-05 |
| mmu-miR-3099 | **-1.21** | 1.34E-05 |  | hsa-miR-132* | **1.19** | 1.83E-03 |
| hsa-miR-720 | **-1.21** | 8.07E-03 |  | mmu-miR-183 | **1.27** | 4.38E-03 |
| hsa-miR-935 | **-1.17** | 1.99E-02 |  | mmu-miR-34b-3p | **1.36** | 4.34E-02 |
| hsa-miR-425* | **-1.12** | 4.90E-03 |  | mmu-miR-27b* | **1.37** | 7.76E-03 |
| mmu-miR-326 | **-1.11** | 1.22E-04 |  | mmu-miR-182 | **1.38** | 1.82E-06 |
| mmu-miR-342-5p | **-1.11** | 2.85E-05 |  | mmu-miR-491 | **1.72** | 3.10E-05 |
| hsa-miR-592 | **-1.10** | 2.10E-02 |  | mmu-miR-34c | **2.05** | 7.73E-05 |
| hsa-miR-363 | **-1.09** | 3.52E-02 |  |  |  |  |
| pri-mmu-mir135b | **-1.08** | 1.29E-03 |  |  |  |  |
| pri-mmu-mir124a-1 | **-1.07** | 7.31E-04 |  |  |  |  |
| mmu-miR-7a | **-1.06** | 4.58E-02 |  |  |  |  |
| hsa-miR-671-3p | **-1.06** | 7.49E-03 |  |  |  |  |
| mmu-miR-301a | **-1.03** | 1.71E-03 |  |  |  |  |
| mmu-miR-128 | **-1.03** | 2.52E-02 |  |  |  |  |
| mmu-miR-93* | **-1.03** | 1.75E-07 |  |  |  |  |
| hsa-miR-92b | **-1.03** | 9.87E-08 |  |  |  |  |
| mmu-miR-378* | **1.00** | 1.84E-02 |  |  |  |  |
| hsa-miR-299-5p | **1.01** | 1.78E-03 |  |  |  |  |
| mmu-miR-3470a | **1.02** | 2.35E-03 |  |  |  |  |
| mmu-miR-669c | **1.02** | 1.59E-03 |  |  |  |  |
| pri-mmu-mir680-2 | **1.04** | 7.74E-03 |  |  |  |  |
| hsa-miR-411* | **1.04** | 2.94E-02 |  |  |  |  |
| mmu-miR-375 | **1.04** | 2.27E-02 |  |  |  |  |
| mmu-miR-216b | **1.04** | 2.86E-04 |  |  |  |  |
| mmu-miR-217 | **1.06** | 2.40E-08 |  |  |  |  |
| mmu-miR-467c | **1.06** | 7.90E-03 |  |  |  |  |
| mmu-miR-1196 | **1.09** | 1.91E-04 |  |  |  |  |
| hsa-let-7d* | **1.12** | 4.14E-02 |  |  |  |  |
| mmu-miR-196a | **1.13** | 6.43E-03 |  |  |  |  |
| mmu-miR-133b | **1.14** | 2.14E-04 |  |  |  |  |
| hsa-miR-10b* | **1.15** | 8.11E-03 |  |  |  |  |
| pri-mmu-mir717 | **1.15** | 2.73E-04 |  |  |  |  |
| hsa-miR-612 | **1.16** | 3.66E-02 |  |  |  |  |
| mmu-miR-127 | **1.17** | 5.91E-12 |  |  |  |  |
| mmu-miR-322* | **1.18** | 1.49E-04 |  |  |  |  |
| mmu-miR-675-3p | **1.19** | 2.25E-06 |  |  |  |  |
| hp_mmu-mir-467h | **1.22** | 4.91E-02 |  |  |  |  |
| hp_mmu-mir-466jx | **1.24** | 2.42E-02 |  |  |  |  |
| mmu-miR-351 | **1.25** | 9.39E-08 |  |  |  |  |
| hp_mmu-mir-466kx | **1.27** | 5.29E-03 |  |  |  |  |
| hsa-miR-217 | **1.27** | 8.90E-03 |  |  |  |  |
| mmu-miR-322 | **1.29** | 4.46E-02 |  |  |  |  |
| hsa-miR-378c | **1.29** | 1.64E-05 |  |  |  |  |
| mmu-miR-574-5p | **1.42** | 3.55E-02 |  |  |  |  |
| mmu-miR-3470b | **1.43** | 7.20E-03 |  |  |  |  |
| hsa-miR-557 | **1.48** | 5.08E-03 |  |  |  |  |
| hp_mmu-mir-466f-4x | **1.52** | 6.47E-03 |  |  |  |  |
| mmu-miR-673-5p | **1.53** | 7.36E-04 |  |  |  |  |
| hsa-miR-937 | **1.53** | 1.02E-04 |  |  |  |  |
| mmu-miR-133a | **1.56** | 1.01E-05 |  |  |  |  |
| hp_mmu-mir-1194 | **1.57** | 2.98E-02 |  |  |  |  |
| hsa-miR-4269 | **1.57** | 4.43E-04 |  |  |  |  |
| hsa-miR-1915* | **1.58** | 7.65E-04 |  |  |  |  |
| mmu-miR-669a | **1.59** | 2.67E-02 |  |  |  |  |
| hsa-miR-2278 | **1.64** | 4.88E-03 |  |  |  |  |
| hp_mmu-mir-466f-3x | **1.68** | 2.85E-02 |  |  |  |  |
| mmu-miR-696 | **1.73** | 4.93E-02 |  |  |  |  |
| hp_mmu-mir-669dx | **1.75** | 3.02E-03 |  |  |  |  |
| mmu-miR-669d | **1.77** | 3.29E-02 |  |  |  |  |
| mmu-miR-15a* | **1.80** | 2.65E-03 |  |  |  |  |
| hsa-miR-3149 | **1.80** | 2.86E-02 |  |  |  |  |
| hsa-miR-422a | **1.82** | 5.73E-04 |  |  |  |  |
| hsa-miR-16-1* | **1.95** | 7.11E-03 |  |  |  |  |
| hsa-miR-1538 | **1.98** | 1.62E-04 |  |  |  |  |
| hsa-miR-595 | **2.02** | 1.47E-02 |  |  |  |  |
| mmu-miR-297 | **2.02** | 2.52E-04 |  |  |  |  |
| hp_hsa-mir-297 | **2.03** | 8.34E-04 |  |  |  |  |
| hsa-miR-1322 | **2.08** | 2.66E-03 |  |  |  |  |
| hp_mmu-mir-297a-6x | **2.10** | 3.45E-02 |  |  |  |  |
| hsa-miR-610 | **2.11** | 6.05E-04 |  |  |  |  |
| mmu-miR-10b | **2.11** | 1.47E-07 |  |  |  |  |
| mmu-miR-1903 | **2.21** | 7.55E-04 |  |  |  |  |
| mmu-miR-467h | **2.21** | 1.52E-02 |  |  |  |  |
| hp_mmu-mir-297a-2 | **2.25** | 1.43E-06 |  |  |  |  |
| hsa-miR-3148 | **2.27** | 2.63E-02 |  |  |  |  |
| hsa-miR-493 | **2.41** | 4.30E-05 |  |  |  |  |
| hsa-miR-16-2* | **2.44** | 6.37E-03 |  |  |  |  |
| mmu-miR-493 | **2.53** | 5.74E-04 |  |  |  |  |
| mmu-miR-145 | **2.73** | 8.98E-06 |  |  |  |  |
| mmu-miR-615-3p | **2.78** | 3.31E-06 |  |  |  |  |
| mmu-miR-126-3p | **2.91** | 3.28E-06 |  |  |  |  |
| mmu-miR-214 | **3.12** | 5.88E-09 |  |  |  |  |
| mmu-miR-199b* | **3.18** | 2.46E-10 |  |  |  |  |
| mmu-miR-199a-5p | **3.29** | 1.15E-06 |  |  |  |  |
| mmu-miR-199b | **3.46** | 1.09E-08 |  |  |  |  |
| mmu-miR-199a-3p | **3.47** | 4.76E-06 |  |  |  |  |
| mmu-miR-143 | **3.56** | 6.35E-06 |  |  |  |  |
| mmu-miR-214* | **4.37** | 7.19E-11 |  |  |  |  |
| mmu-miR-10a | **4.58** | 5.98E-09 |  |  |  |  |
| mmu-miR-206 | **6.64** | 2.06E-09 |  |  |  |  |

^*^ Mmu – mouse musculus. Hsa- homo sapiens, hp – hairpin, pri – primary.
