# Supplementary material for: MicroRNA Profiling as Tool for In Vitro Developmental Neurotoxicity Testing: The Case of Sodium Valproate
Source: PLoS One. 2014 Jun 4;9(6):e98892. doi: 10.1371/journal.pone.0098892 (PMC4045889; doi:10.1371/journal.pone.0098892)
Supplement: Table S3 — Valproate and arsenite intersected miRNA. (DOCX) [file pone.0098892.s008.docx]

Table S3: Valproate and arsenite intersected miRNA.

| **Name** | **Arsenite: log_2_(FC)** | **Valproate:**  **log_2_(FC)** |
| --- | --- | --- |
| mmu-miR-491 | 1.72 | -1.98 |
| mmu-miR-383 | -1.01 | -2.05 |
| hsa-miR-383 | -1.09 | -2.01 |
| mmu-miR-214 | -1.24 | 3.12 |
| mmu-miR-199b | -1.43 | 3.46 |
| mmu-miR-199a-5p | -1.25 | 3.29 |
| mmu-miR-199a-3p | -1.54 | 3.47 |
| mmu-miR-145 | -1.02 | 12.70 |
| hsa-miR-2278 | -1.44 | 1.64 |
| hp_mmu-mir-466f-4x | -1.30 | 1.52 |
