# Supplementary material for: MicroRNA Profiling as Tool for In Vitro Developmental Neurotoxicity Testing: The Case of Sodium Valproate
Source: PLoS One. 2014 Jun 4;9(6):e98892. doi: 10.1371/journal.pone.0098892 (PMC4045889; doi:10.1371/journal.pone.0098892)
Supplement: Table S4 — Genes, responding to valproate treatment during neural differentiation of ESCs. Log2 of the mean fold change for each mRNA normalized to untreated control is given. A total of 377 mRNA were identified differently expressed in neurally differentiating mES cells under VPA treatment (300 µM) compared to untreated control during 16 days of neural differentiation. Threshold was set as over 2-fold change and p<0.05. (DOCX) [file pone.0098892.s009.docx]

**Table S4: Genes, responding to valproate treatment during neural differentiation of ESCs.**

| **Gene Symbol** | **log_2_(FC)** | p-value | RefSeq |
| --- | --- | --- | --- |
| Rspo2 | **-2,46** | 7,3E-04 | NM_172815 |
| Cbln2 | **-2,17** | 1,3E-05 | NM_172633 |
| Wnt8b | **-2,13** | 6,0E-05 | NM_011720 |
| Sphkap | **-2,06** | 2,1E-06 | NM_172430 |
| Lgi1 | **-2,03** | 6,4E-05 | NM_020278 |
| Eomes | **-1,95** | 7,2E-05 | NM_010136 |
| Ttr | **-1,90** | 1,3E-03 | NM_013697 |
| Zic4 | **-1,88** | 6,0E-05 | NM_009576 |
| Dmbx1 | **-1,84** | 3,1E-05 | NM_130865 |
| Rasgrp1 | **-1,76** | 3,1E-04 | NM_011246 |
| Mir135a-2 | **-1,75** | 2,9E-05 | NR_029812 |
| Kcnd2 | **-1,71** | 1,1E-04 | NM_019697 |
| A230006K03Rik | **-1,70** | 9,6E-04 | DQ656357 |
| Gabra1 | **-1,67** | 1,0E-04 | NM_010250 |
| Cntn6 | **-1,66** | 1,2E-04 | NM_017383 |
| A930017M01Rik | **-1,66** | 2,5E-05 | NR_033609 |
| Rspo3 | **-1,65** | 2,3E-03 | NM_028351 |
| Amy1 | **-1,63** | 2,2E-05 | NM_007446 |
| Unc5d | **-1,61** | 4,9E-05 | NM_153135 |
| Otx1 | **-1,60** | 5,9E-04 | NM_011023 |
| Mir384 | **-1,60** | 1,0E-03 | NR_029910 |
| 1500035N22Rik | **-1,58** | 2,2E-04 | ENSMUST00000075081 |
| C130030K03Rik | **-1,57** | 6,1E-04 | AK048022 |
| 2900055J20Rik | **-1,52** | 3,9E-04 | ENSMUST00000096572 |
| Gria1 | **-1,51** | 6,1E-06 | NM_001113325 |
| Glra2 | **-1,50** | 3,9E-05 | NM_183427 |
| March1 | **-1,48** | 8,2E-05 | NM_175188 |
| Gad2 | **-1,48** | 9,8E-05 | NM_008078 |
| St6gal2 | **-1,48** | 2,1E-05 | NM_172829 |
| Fam163a | **-1,47** | 1,1E-03 | NM_177838 |
| Grm5 | **-1,47** | 4,7E-05 | NM_001143834 |
| Gad1 | **-1,47** | 3,8E-05 | NM_008077 |
| Lrfn5 | **-1,46** | 3,8E-05 | NM_178714 |
| Trhr | **-1,46** | 3,2E-03 | NM_013696 |
| Odz1 | **-1,45** | 5,0E-05 | NM_011855 |
| Ralyl | **-1,42** | 3,8E-05 | NM_178631 |
| Grm1 | **-1,42** | 6,6E-04 | ENSMUST00000044306 |
| 4930412O13Rik | **-1,42** | 6,2E-05 | NR_024257 |
| Zic5 | **-1,42** | 5,1E-04 | NM_022987 |
| Gabrr1 | **-1,41** | 1,3E-03 | ENSMUST00000029947 |
| Tcfap2b | **-1,39** | 2,8E-05 | NM_009334 |
| Vsnl1 | **-1,38** | 6,5E-04 | NM_012038 |
| Tcfap2d | **-1,36** | 8,1E-04 | NM_153154 |
| Cpm | **-1,35** | 5,2E-04 | NM_027468 |
| 1700084C01Rik | **-1,35** | 7,0E-04 | BC150846 |
| Lrp1b | **-1,34** | 3,1E-04 | NM_053011 |
| Gm9911 | **-1,32** | 4,2E-03 | AK035883 |
| Slc27a2 | **-1,32** | 9,4E-05 | NM_011978 |
| Gabrg2 | **-1,31** | 2,6E-04 | NM_008073 |
| Pax7 | **-1,30** | 1,1E-04 | NM_011039 |
| Dscam | **-1,29** | 2,6E-04 | NM_031174 |
| Vstm2a | **-1,28** | 4,5E-05 | NM_145967 |
| Sox14 | **-1,28** | 1,1E-04 | NM_011440 |
| Omg | **-1,27** | 3,8E-05 | NM_019409 |
| Stx3 | **-1,26** | 9,6E-05 | NM_001025307 |
| Fezf2 | **-1,25** | 2,8E-04 | NM_080433 |
| Slc44a5 | **-1,24** | 4,4E-04 | NM_001081263 |
| Pgap1 | **-1,24** | 6,6E-04 | ENSMUST00000097739 |
| Otx2 | **-1,24** | 1,1E-03 | NM_144841 |
| Ovgp1 | **-1,24** | 2,1E-05 | NM_007696 |
| Tal2 | **-1,23** | 4,8E-03 | NM_009317 |
| Zic3 | **-1,21** | 1,5E-03 | NM_009575 |
| Fam5c | **-1,21** | 1,4E-04 | NM_153539 |
| AI504432 | **-1,21** | 1,3E-04 | NR_033498 |
| Zfpm2 | **-1,21** | 3,0E-04 | NM_011766 |
| Pou6f2 | **-1,20** | 1,5E-04 | NM_175006 |
| En2 | **-1,18** | 3,7E-05 | NM_010134 |
| Cyp4f15 | **-1,17** | 8,3E-05 | NM_134127 |
| Serpinb1a | **-1,17** | 5,7E-04 | NM_025429 |
| Cdh12 | **-1,16** | 2,5E-04 | NM_001008420 |
| B3galt2 | **-1,16** | 1,5E-05 | NM_020025 |
| Calml4 | **-1,15** | 5,7E-03 | NM_138304 |
| Ndst3 | **-1,14** | 9,6E-05 | NM_031186 |
| Barhl2 | **-1,14** | 4,4E-05 | NM_001005477 |
| Cnpy1 | **-1,14** | 7,8E-04 | NM_175651 |
| Rgs2 | **-1,13** | 8,4E-05 | NM_009061 |
| Grm8 | **-1,12** | 1,5E-04 | NM_008174 |
| Slc7a11 | **-1,12** | 4,5E-03 | NM_011990 |
| Gjd2 | **-1,12** | 3,2E-03 | NM_010290 |
| Cacng3 | **-1,12** | 1,8E-03 | NM_019430 |
| Nkain2 | **-1,11** | 1,2E-03 | NM_001013411 |
| Spock3 | **-1,11** | 2,0E-03 | NM_023689 |
| Frmd3 | **-1,11** | 6,9E-06 | NM_172869 |
| Helt | **-1,10** | 3,3E-04 | NM_173789 |
| Spry1 | **-1,10** | 1,1E-05 | NM_011896 |
| Lmo1 | **-1,10** | 4,4E-05 | NM_057173 |
| Chrm3 | **-1,09** | 5,4E-06 | NM_033269 |
| Pcdh11x | **-1,09** | 2,2E-04 | NM_001081385 |
| Ccdc85a | **-1,09** | 8,9E-05 | NM_181577 |
| Mir135b | **-1,08** | 1,3E-03 | NR_029777 |
| Olig1 | **-1,07** | 1,6E-03 | NM_016968 |
| Ano1 | **-1,07** | 5,4E-04 | NM_178642 |
| Gm9900 | **-1,07** | 3,7E-04 | ENSMUST00000065533 |
| Mir124a-1 | **-1,07** | 7,3E-04 | NR_029813 |
| Ptchd1 | **-1,07** | 3,8E-06 | NM_001093750 |
| Trpm3 | **-1,06** | 4,1E-05 | NM_001035244 |
| Gabrb2 | **-1,06** | 5,5E-04 | NM_008070 |
| Lhx1 | **-1,06** | 1,1E-04 | NM_008498 |
| Ddn | **-1,05** | 4,3E-04 | NM_001013741 |
| C230004F18Rik | **-1,05** | 8,4E-05 | NR_030706 |
| Trhde | **-1,04** | 2,8E-04 | NM_146241 |
| Gm9958 | **-1,04** | 2,9E-04 | ENSMUST00000068250 |
| 3110082D06Rik | **-1,03** | 2,0E-04 | NM_028474 |
| Gata3 | **-1,02** | 4,2E-03 | NM_008091 |
| Clvs2 | **-1,02** | 3,7E-03 | NM_175448 |
| Slc17a6 | **-1,02** | 1,8E-03 | NM_080853 |
| Lypd1 | **-1,01** | 4,9E-06 | NM_145100 |
| Phyhd1 | **-1,01** | 3,6E-04 | NM_172267 |
| Npnt | **-1,01** | 2,4E-04 | NM_033525 |
| Gabra2 | **-1,00** | 4,4E-04 | NM_008066 |
| Copz2 | **1,00** | 7,4E-05 | NM_019877 |
| Bnc2 | **1,00** | 4,0E-04 | NM_172870 |
| Fam111a | **1,00** | 1,3E-03 | BC038020 |
| Ssty1 | **1,00** | 1,6E-02 | NM_009220 |
| Hist1h1a | **1,01** | 8,8E-05 | NM_030609 |
| Lsp1 | **1,01** | 3,7E-04 | NM_019391 |
| Plagl1 | **1,01** | 1,4E-04 | NM_009538 |
| Gpr17 | **1,01** | 7,5E-04 | NM_001025381 |
| Sec24d | **1,01** | 4,8E-05 | NM_027135 |
| Arpc1b | **1,01** | 3,3E-05 | NM_023142 |
| Nid1 | **1,02** | 9,9E-04 | NM_010917 |
| Fbln1 | **1,02** | 5,9E-04 | NM_010180 |
| Tmem26 | **1,03** | 7,1E-04 | NM_177794 |
| Irgm1 | **1,03** | 3,7E-03 | NM_008326 |
| Frzb | **1,03** | 1,8E-03 | NM_011356 |
| Mir680-2 | **1,04** | 7,7E-03 | NR_030448 |
| Ptch1 | **1,04** | 2,7E-03 | NM_008957 |
| Ehd2 | **1,04** | 6,1E-04 | NM_153068 |
| Fignl1 | **1,04** | 5,8E-04 | NM_001163359 |
| Itga5 | **1,05** | 3,1E-03 | NM_010577 |
| Ror2 | **1,05** | 4,8E-04 | NM_013846 |
| Dkk3 | **1,05** | 1,9E-03 | NM_015814 |
| Snx7 | **1,05** | 2,2E-04 | NM_029655 |
| Lox | **1,05** | 6,5E-04 | NM_010728 |
| Olfr1333 | **1,06** | 3,2E-02 | NM_207157 |
| Csprs | **1,06** | 1,6E-02 | NM_033616 |
| Tpm2 | **1,06** | 2,6E-04 | NM_009416 |
| Pdlim3 | **1,06** | 4,5E-04 | NM_016798 |
| Layn | **1,06** | 5,1E-05 | NM_001033534 |
| LOC665746 | **1,06** | 6,9E-03 | ENSMUST00000115889 |
| Ddx58 | **1,07** | 1,2E-05 | NM_172689 |
| Car13 | **1,07** | 7,5E-04 | NM_024495 |
| Net1 | **1,07** | 1,7E-04 | NM_019671 |
| Fbxl7 | **1,07** | 2,3E-03 | BC050864 |
| Ltbp1 | **1,07** | 8,0E-04 | NM_019919 |
| Crhbp | **1,07** | 8,9E-05 | NM_198408 |
| Gpr126 | **1,07** | 1,0E-04 | NM_001002268 |
| Gm3994 | **1,07** | 9,8E-03 | ENSMUST00000098805 |
| Vmn2r43 | **1,07** | 2,2E-02 | NM_198961 |
| Crybg3 | **1,07** | 8,0E-05 | NM_174848 |
| Lamb1-1 | **1,08** | 1,7E-04 | NM_008482 |
| Emilin2 | **1,08** | 7,2E-05 | NM_145158 |
| Ngfr | **1,08** | 4,6E-03 | NM_033217 |
| Itih5 | **1,08** | 2,3E-03 | NM_172471 |
| Fbn2 | **1,08** | 5,5E-05 | NM_010181 |
| Antxr2 | **1,08** | 1,4E-04 | NM_133738 |
| Cd93 | **1,08** | 9,4E-04 | NM_010740 |
| Arhgap29 | **1,08** | 1,2E-03 | NM_172525 |
| 9030625A04Rik | **1,08** | 1,1E-05 | BC116748 |
| Vcl | **1,09** | 3,4E-05 | NM_009502 |
| Ptges | **1,09** | 5,5E-05 | NM_022415 |
| Col1a1 | **1,09** | 8,7E-05 | NM_007742 |
| Cyr61 | **1,09** | 4,5E-05 | NM_010516 |
| A530040E14Rik | **1,09** | 3,1E-02 | BC100303 |
| Cyp39a1 | **1,10** | 9,5E-04 | NM_018887 |
| Pdgfrb | **1,10** | 2,7E-03 | NM_001146268 |
| Tgfbr3 | **1,10** | 1,8E-03 | NM_011578 |
| BC028528 | **1,11** | 1,5E-03 | BC028528 |
| Sall1 | **1,11** | 3,9E-03 | NM_021390 |
| Trim25 | **1,11** | 4,5E-03 | NM_009546 |
| Colec12 | **1,11** | 6,1E-05 | NM_130449 |
| Twist1 | **1,12** | 7,6E-05 | NM_011658 |
| Arhgdib | **1,12** | 1,7E-03 | NM_007486 |
| Got1l1 | **1,12** | 3,4E-04 | NM_029674 |
| S100a11 | **1,12** | 1,1E-04 | NM_016740 |
| Adh1 | **1,12** | 4,5E-04 | NM_007409 |
| Foxd1 | **1,12** | 2,3E-03 | NM_008242 |
| Sema3d | **1,13** | 5,6E-04 | NM_028882 |
| Sulf1 | **1,13** | 5,7E-04 | NM_172294 |
| Hoxb7 | **1,13** | 8,6E-04 | NM_010460 |
| Slit3 | **1,13** | 3,1E-03 | NM_011412 |
| Ucp2 | **1,13** | 8,8E-04 | NM_011671 |
| Tiam2 | **1,13** | 1,2E-02 | NM_001122998 |
| Smoc2 | **1,14** | 5,7E-04 | NM_022315 |
| Lpar4 | **1,14** | 4,3E-04 | NM_175271 |
| Mir717 | **1,15** | 2,7E-04 | NR_030497 |
| Fam101b | **1,15** | 1,0E-02 | NM_029658 |
| Ccdc122 | **1,15** | 3,7E-04 | NM_175369 |
| Lhfpl2 | **1,15** | 2,3E-03 | NM_172589 |
| LOC100041256 | **1,15** | 3,7E-02 | BC089466 |
| F11r | **1,16** | 7,1E-04 | NM_172647 |
| Kdelr3 | **1,16** | 1,8E-03 | NM_134090 |
| Bche | **1,16** | 7,6E-04 | NM_009738 |
| Cyp1b1 | **1,16** | 6,4E-04 | NM_009994 |
| Fzd4 | **1,17** | 2,8E-05 | NM_008055 |
| Casp8 | **1,17** | 1,9E-03 | NM_009812 |
| C330024D21Rik | **1,17** | 6,8E-03 | NR_015582 |
| Ang | **1,17** | 1,6E-06 | NM_001161731 |
| Ube1y1 | **1,18** | 3,6E-04 | NM_011667 |
| T | **1,18** | 1,5E-03 | NM_009309 |
| Meox2 | **1,18** | 9,6E-04 | NM_008584 |
| Rbm46 | **1,19** | 1,4E-03 | NM_001146328 |
| Hoxc4 | **1,19** | 3,3E-03 | NM_013553 |
| A430107O13Rik | **1,19** | 6,4E-04 | BC151018 |
| Vmn2r37 | **1,20** | 9,6E-03 | NM_009489 |
| Ahr | **1,20** | 4,4E-04 | NM_013464 |
| Sfrp2 | **1,20** | 1,6E-05 | NM_009144 |
| Stard13 | **1,21** | 5,4E-04 | NM_001163493 |
| Sncg | **1,21** | 5,1E-05 | NM_011430 |
| Olfr1381 | **1,21** | 3,2E-02 | NM_146469 |
| Cybrd1 | **1,21** | 1,3E-05 | NM_028593 |
| Chodl | **1,22** | 8,2E-05 | NM_139134 |
| Pdgfrl | **1,23** | 1,1E-04 | NM_026840 |
| Itga9 | **1,23** | 3,8E-04 | NM_133721 |
| 1810011O10Rik | **1,23** | 1,7E-04 | NM_026931 |
| Anxa5 | **1,23** | 8,7E-04 | NM_009673 |
| Tmem119 | **1,24** | 5,6E-06 | NM_146162 |
| Tgfbi | **1,24** | 1,9E-03 | NM_009369 |
| LOC100039753 | **1,24** | 3,2E-02 | NM_001017394 |
| LOC100041992 | **1,24** | 1,2E-02 | ENSMUST00000115910 |
| Nefm | **1,24** | 1,2E-05 | NM_008691 |
| Parm1 | **1,24** | 7,1E-05 | NM_145562 |
| Tmem45a | **1,24** | 6,9E-04 | NM_019631 |
| Col12a1 | **1,25** | 9,8E-05 | NM_007730 |
| Hpse | **1,25** | 1,1E-03 | NM_152803 |
| Itm2a | **1,26** | 4,7E-04 | NM_008409 |
| Agtr2 | **1,26** | 2,3E-03 | NM_007429 |
| Col1a2 | **1,26** | 2,5E-05 | NM_007743 |
| Dlc1 | **1,27** | 1,4E-03 | NM_015802 |
| Fam115c | **1,27** | 6,4E-06 | BC011487 |
| 5730596B20Rik | **1,27** | 1,1E-03 | ENSMUST00000070587 |
| Pde8a | **1,27** | 1,8E-03 | NM_008803 |
| Fam38a | **1,27** | 1,3E-04 | NM_001037298 |
| Tgfbr2 | **1,28** | 1,2E-04 | NM_009371 |
| Dock6 | **1,28** | 5,4E-03 | NM_177030 |
| Timp3 | **1,29** | 4,8E-03 | NM_011595 |
| Hoxb5 | **1,29** | 2,2E-03 | NM_008268 |
| Snai2 | **1,29** | 2,9E-04 | NM_011415 |
| Emcn | **1,30** | 1,2E-03 | NM_001163522 |
| Zc3hav1 | **1,30** | 4,3E-04 | NM_028421 |
| Gxylt2 | **1,30** | 2,4E-04 | NM_198612 |
| Fmr1nb | **1,30** | 4,1E-04 | NM_174993 |
| Sema5a | **1,31** | 7,1E-06 | NM_009154 |
| Stra6 | **1,32** | 5,9E-04 | NM_009291 |
| LOC382133 | **1,32** | 3,1E-02 | BC049586 |
| Tpbpa | **1,32** | 4,9E-03 | NM_009411 |
| Dkk2 | **1,32** | 2,0E-04 | NM_020265 |
| Gm7609 | **1,32** | 2,4E-02 | NM_001081746 |
| G730007D18Rik | **1,33** | 2,6E-04 | AK144596 |
| Mbnl3 | **1,34** | 6,7E-04 | NM_134163 |
| Hoxd4 | **1,34** | 9,5E-03 | NM_010469 |
| Mc5r | **1,34** | 4,8E-05 | NM_013596 |
| LOC665448 | **1,34** | 7,6E-03 | XR_035752 |
| Olfml3 | **1,34** | 1,6E-04 | NM_133859 |
| Tbx2 | **1,35** | 2,2E-03 | NM_009324 |
| Pcolce | **1,35** | 9,3E-04 | NM_008788 |
| Hoxd8 | **1,36** | 1,1E-03 | NM_008276 |
| Vmn2r42 | **1,36** | 8,4E-03 | NM_009493 |
| Airn | **1,36** | 1,7E-04 | NR_002853 |
| Egflam | **1,36** | 3,1E-04 | NM_178748 |
| Igfbp4 | **1,37** | 2,8E-04 | NM_010517 |
| Bmp5 | **1,37** | 8,7E-04 | NM_007555 |
| Hoxc5 | **1,37** | 5,4E-03 | NM_175730 |
| Igfbp3 | **1,38** | 4,7E-04 | NM_008343 |
| 2810047C21Rik1 | **1,38** | 1,9E-04 | NR_015598 |
| Hs3st2 | **1,39** | 2,2E-03 | NM_001081327 |
| Hspb1 | **1,40** | 6,8E-03 | NM_013560 |
| Hoxa3 | **1,41** | 2,1E-03 | NM_010452 |
| Hmga2 | **1,41** | 4,9E-05 | NM_010441 |
| Figf | **1,41** | 4,9E-04 | NM_010216 |
| Adamts1 | **1,41** | 1,3E-04 | NM_009621 |
| Dlx1 | **1,41** | 1,5E-04 | NM_010053 |
| Tagln2 | **1,41** | 1,8E-04 | NM_178598 |
| Six1 | **1,41** | 8,9E-04 | NM_009189 |
| Chrna5 | **1,41** | 4,0E-06 | NM_176844 |
| Vmn2r34 | **1,42** | 6,7E-03 | NM_001105066 |
| LOC434960 | **1,42** | 2,7E-02 | BC089498 |
| MGC107098 | **1,42** | 2,8E-02 | BC089465 |
| Lrig3 | **1,43** | 1,4E-04 | NM_177152 |
| Serpinf1 | **1,43** | 1,0E-04 | NM_011340 |
| LOC665346 | **1,44** | 1,3E-02 | ENSMUST00000115954 |
| Ifitm3 | **1,45** | 3,2E-04 | NM_025378 |
| Ntn1 | **1,46** | 1,0E-02 | NM_008744 |
| Thy1 | **1,47** | 7,6E-04 | NM_009382 |
| Tgfb3 | **1,48** | 2,2E-04 | NM_009368 |
| Gpr124 | **1,48** | 6,6E-05 | NM_054044 |
| Ror1 | **1,48** | 1,1E-04 | NM_013845 |
| Zfp772 | **1,49** | 1,1E-04 | BC023179 |
| Vtn | **1,50** | 3,9E-02 | NM_011707 |
| Mfap4 | **1,50** | 1,0E-04 | NM_029568 |
| Hoxa5 | **1,50** | 8,6E-03 | NM_010453 |
| Fap | **1,50** | 2,9E-04 | NM_007986 |
| Vmn2r50 | **1,50** | 4,6E-05 | ENSMUST00000086298 |
| Tnfaip6 | **1,51** | 2,7E-05 | NM_009398 |
| Fn1 | **1,52** | 2,0E-04 | NM_010233 |
| Ssty2 | **1,52** | 2,4E-02 | NM_023546 |
| Vmn2r51 | **1,53** | 1,9E-02 | NM_001105179 |
| Slc40a1 | **1,54** | 4,6E-04 | NM_016917 |
| Col6a3 | **1,54** | 1,5E-04 | AF064749 |
| Pdgfra | **1,54** | 2,4E-05 | NM_011058 |
| Emp1 | **1,55** | 4,8E-05 | NM_010128 |
| Tbx15 | **1,55** | 8,1E-05 | NM_009323 |
| Lgals1 | **1,56** | 6,2E-05 | NM_008495 |
| Arhgap28 | **1,58** | 2,9E-04 | NM_172964 |
| Col3a1 | **1,59** | 5,1E-05 | NM_009930 |
| Fam198b | **1,60** | 5,1E-05 | NM_133187 |
| Erbb3 | **1,62** | 3,0E-05 | NM_010153 |
| S1pr3 | **1,64** | 1,9E-04 | NM_010101 |
| Dlk1 | **1,65** | 1,2E-04 | NM_010052 |
| Emp3 | **1,65** | 2,6E-05 | NM_010129 |
| Lpar1 | **1,65** | 1,5E-05 | NM_010336 |
| Fndc3c1 | **1,66** | 2,7E-04 | NM_001007580 |
| Mybpc1 | **1,66** | 4,3E-05 | NM_175418 |
| Itga8 | **1,66** | 1,5E-04 | NM_001001309 |
| Cdh19 | **1,66** | 7,7E-05 | NM_001081386 |
| Npy | **1,67** | 6,1E-05 | NM_023456 |
| Hic1 | **1,67** | 2,1E-03 | NM_010430 |
| Hoxc8 | **1,68** | 5,5E-04 | NM_010466 |
| Hoxa2 | **1,70** | 8,7E-05 | NM_010451 |
| Capn6 | **1,70** | 1,6E-05 | NM_007603 |
| A930038C07Rik | **1,70** | 2,8E-04 | NM_172399 |
| Sim1 | **1,70** | 2,2E-04 | NM_011376 |
| Elf4 | **1,72** | 1,4E-03 | NM_019680 |
| Hoxb9 | **1,72** | 3,0E-03 | NM_008270 |
| Islr | **1,73** | 1,7E-04 | NM_012043 |
| Crabp1 | **1,74** | 2,1E-05 | NM_013496 |
| Lama4 | **1,76** | 6,7E-05 | NM_010681 |
| Myh3 | **1,77** | 1,0E-03 | NM_001099635 |
| Lrrc17 | **1,80** | 1,6E-04 | NM_028977 |
| Tcfl5 | **1,82** | 2,5E-05 | NM_178254 |
| Glra1 | **1,82** | 7,6E-04 | NM_020492 |
| Acta2 | **1,85** | 6,0E-04 | NM_007392 |
| Fibin | **1,85** | 1,1E-05 | NM_026271 |
| Dab2 | **1,85** | 4,0E-05 | NM_023118 |
| Igf2 | **1,87** | 4,9E-05 | NM_001122737 |
| Zfp125 | **1,88** | 1,6E-05 | ENSMUST00000079237 |
| Prrx1 | **1,90** | 1,7E-05 | NM_175686 |
| Adamts9 | **1,90** | 3,3E-05 | NM_175314 |
| Ddr2 | **1,93** | 1,7E-05 | NM_022563 |
| Car3 | **1,95** | 1,1E-03 | NM_007606 |
| S100a10 | **1,96** | 6,7E-06 | NM_009112 |
| Dnm3os | **1,97** | 2,8E-05 | NR_002870 |
| Cdh5 | **1,98** | 8,3E-04 | NM_009868 |
| Hoxb2 | **2,01** | 1,4E-03 | NM_134032 |
| Nrk | **2,06** | 1,2E-04 | NM_013724 |
| Angptl1 | **2,09** | 1,9E-05 | NM_028333 |
| Gm10664 | **2,10** | 2,1E-03 | ENSMUST00000098718 |
| Hoxb4 | **2,13** | 3,3E-04 | NM_010459 |
| Egfl6 | **2,15** | 4,2E-06 | NM_019397 |
| Tbx10 | **2,15** | 4,6E-05 | NM_001001320 |
| Gpx3 | **2,16** | 1,3E-03 | NM_001083929 |
| Cd34 | **2,19** | 2,3E-05 | NM_001111059 |
| Gm1564 | **2,21** | 7,5E-05 | NM_001127576 |
| Ednra | **2,22** | 3,4E-06 | NM_010332 |
| Myl4 | **2,22** | 9,5E-04 | NM_010858 |
| Npr3 | **2,26** | 5,4E-05 | NM_008728 |
| Hoxc6 | **2,26** | 8,4E-04 | NM_010465 |
| Dcn | **2,28** | 5,8E-05 | NM_007833 |
| Gpc3 | **2,28** | 1,4E-05 | NM_016697 |
| Osr1 | **2,29** | 9,0E-06 | NM_011859 |
| Lum | **2,29** | 1,3E-04 | NM_008524 |
| Gm12569 | **2,30** | 1,1E-04 | ENSMUST00000074198 |
| Myl1 | **2,31** | 1,7E-03 | NM_021285 |
| Ogn | **2,32** | 7,9E-06 | NM_008760 |
| Efemp1 | **2,34** | 3,3E-05 | NM_146015 |
| Slc38a4 | **2,35** | 1,5E-06 | NM_027052 |
| Moxd1 | **2,40** | 1,7E-05 | NM_021509 |
| H19 | **2,45** | 3,7E-04 | NR_001592 |
| Igf1 | **2,53** | 2,1E-06 | NM_010512 |
| Hoxb3 | **2,59** | 4,6E-04 | NM_001079869 |
| Crabp2 | **2,61** | 8,5E-06 | NM_007759 |
| Cnn2 | **2,64** | 3,7E-05 | NM_007725 |
| Postn | **2,66** | 1,1E-04 | NM_015784 |
| Hoxb8 | **2,79** | 4,2E-06 | NM_010461 |
| Zcchc5 | **2,80** | 2,5E-05 | NM_199468 |
| Aspn | **2,86** | 1,3E-04 | NM_025711 |
| Actc1 | **2,93** | 3,5E-04 | NM_009608 |
