# Supplementary material for: MicroRNA Profiling as Tool for In Vitro Developmental Neurotoxicity Testing: The Case of Sodium Valproate
Source: PLoS One. 2014 Jun 4;9(6):e98892. doi: 10.1371/journal.pone.0098892 (PMC4045889; doi:10.1371/journal.pone.0098892)
Supplement: Table S5 — miRNAs and their target mRNAs, responding to valproate treatment in a reciprocal manner during neural differentiation of ESCs. log2 of the mean fold change for each miRNA and mRNA normalized to untreated control is given. (DOCX) [file pone.0098892.s010.docx]

**Table S5: miRNAs and their target mRNAs, responding to valproate treatment at reciprocal manner during neural differentiation of ESCs.**

| **miRNA symbol** | **log_2_(FC)** | **Gene symbol** | **log_2_(FC)** | **Confidence** | **Source** |
| --- | --- | --- | --- | --- | --- |
| mmu-miR-128 | **-1.03** | ACTA2 | **1.85** | HP | TargetScan |
| mmu-miR-128 | **-1.03** | ARHGAP28 | **1.58** | HP | TargetScan |
| mmu-miR-128 | **-1.03** | C8orf4 | **1.23** | HP | TargetScan |
| mmu-miR-128 | **-1.03** | CDH5 | **1.98** | HP | TargetScan |
| mmu-miR-128 | **-1.03** | CYP39A1 | **1.10** | HP | TargetScan |
| mmu-miR-128 | **-1.03** | DKK2 | **1.32** | HP | TargetScan |
| mmu-miR-128 | **-1.03** | GPR126 | **1.07** | HP | TargetScan |
| mmu-miR-128 | **-1.03** | HIC1 | **1.67** | HP | TargetScan |
| mmu-miR-128 | **-1.03** | HOXB3 | **2.59** | HP | TargetScan |
| mmu-miR-128 | **-1.03** | HOXB8 | **2.79** | HP | TargetScan |
| mmu-miR-128 | **-1.03** | HOXC6 | **2.26** | HP | TargetScan |
| mmu-miR-128 | **-1.03** | IGF1 | **2.53** | HP | TargetScan |
| mmu-miR-128 | **-1.03** | ITGA5 | **1.05** | HP | TargetScan |
| mmu-miR-128 | **-1.03** | NGFR | **1.08** | HP | TargetScan |
| mmu-miR-128 | **-1.03** | NRK | **2.06** | HP | TargetScan |
| mmu-miR-128 | **-1.03** | PDGFRA | **1.54** | HP | TargetScan |
| mmu-miR-128 | **-1.03** | PTCH1 | **1.04** | HP | TargetScan |
| mmu-miR-301a | **-1.03** | CA13 | **1.07** | HP | TargetScan |
| mmu-miR-301a | **-1.03** | COL6A3 | **1.54** | HP | TargetScan |
| mmu-miR-301a | **-1.03** | DLC1 | **1.27** | HP | TargetScan |
| mmu-miR-301a | **-1.03** | ERBB3 | **1.62** | HP | TargetScan |
| mmu-miR-301a | **-1.03** | FIBIN | **1.85** | HP | TargetScan |
| mmu-miR-301a | **-1.03** | FRZB | **1.03** | HP | TargetScan |
| mmu-miR-301a | **-1.03** | HOXA3 | **1.41** | HP | TargetScan |
| mmu-miR-301a | **-1.03** | HOXA5 | **1.50** | EO | TarBase,TargetScan,miRecords |
| mmu-miR-301a | **-1.03** | HOXB3 | **2.59** | HP | TargetScan |
| mmu-miR-301a | **-1.03** | IGF1 | **2.53** | HP | TargetScan |
| mmu-miR-301a | **-1.03** | MEOX2 | **1.18** | EO | TarBase,TargetScan,miRecords |
| mmu-miR-301a | **-1.03** | PDGFRA | **1.54** | HP | TargetScan |
| mmu-miR-301a | **-1.03** | SMOC2 | **1.14** | HP | TargetScan |
| mmu-miR-301a | **-1.03** | STARD13 | **1.21** | HP | TargetScan |
| mmu-miR-301a | **-1.03** | SULF1 | **1.13** | HP | TargetScan |
| mmu-miR-301a | **-1.03** | TGFBR2 | **1.28** | HP | TargetScan |
| mmu-miR-7a | **-1.06** | ACTC1 | **2.93** | HP | TargetScan |
| mmu-miR-7a | **-1.06** | COL1A2 | **1.26** | HP | TargetScan |
| mmu-miR-7a | **-1.06** | COLEC12 | **1.11** | HP | TargetScan |
| mmu-miR-7a | **-1.06** | CRYBG3 | **1.08** | HP | TargetScan |
| mmu-miR-7a | **-1.06** | FBXL7 | **1.07** | HP | TargetScan |
| mmu-miR-7a | **-1.06** | FIGF | **1.41** | HP | TargetScan |
| mmu-miR-7a | **-1.06** | GPR124 | **1.48** | HP | TargetScan |
| mmu-miR-7a | **-1.06** | ITGA9 | **1.23** | HP | TargetScan |
| mmu-miR-7a | **-1.06** | NEFM | **1.24** | EO | Ingenuity Expert Findings |
| mmu-miR-7a | **-1.06** | SLC38A4 | **2.35** | HP | TargetScan |
| mmu-miR-7a | **-1.06** | TMEM26 | **1.03** | HP | TargetScan |
| mmu-miR-7a | **-1.06** | VCL | **1.09** | HP | TargetScan |
| mmu-mir-124 | **-1.07** | ADAMTS9 | **1.90** | HP | TargetScan |
| mmu-mir-124 | **-1.07** | AHR | **1.20** | EO | TarBase,TargetScan,miRecords |
| mmu-mir-124 | **-1.07** | ANGPTL1 | **2.09** | HP | TargetScan |
| mmu-mir-124 | **-1.07** | ANTXR2 | **1.08** | HP | TargetScan |
| mmu-mir-124 | **-1.07** | ANXA5 | **1.23** | HP | TargetScan |
| mmu-mir-124 | **-1.07** | ARHGAP28 | **1.58** | HP | TargetScan |
| mmu-mir-124 | **-1.07** | ARHGAP29 | **1.08** | EO | TarBase,miRecords |
| mmu-mir-124 | **-1.07** | ARPC1B | **1.01** | EO | TarBase,TargetScan,miRecords |
| mmu-mir-124 | **-1.07** | CAPN6 | **1.70** | HP | TargetScan |
| mmu-mir-124 | **-1.07** | CHODL | **1.22** | EO | TargetScan,miRecords |
| mmu-mir-124 | **-1.07** | COL12A1 | **1.25** | HP | TargetScan |
| mmu-mir-124 | **-1.07** | CRYBG3 | **1.08** | HP | TargetScan |
| mmu-mir-124 | **-1.07** | CYP1B1 | **1.16** | EO | TarBase,miRecords |
| mmu-mir-124 | **-1.07** | ELF4 | **1.72** | EO | TarBase,TargetScan,miRecords |
| mmu-mir-124 | **-1.07** | ERBB3 | **1.62** | HP | TargetScan |
| mmu-mir-124 | **-1.07** | F11R | **1.16** | EO | TarBase,TargetScan,miRecords |
| mmu-mir-124 | **-1.07** | FZD4 | **1.17** | HP | TargetScan |
| mmu-mir-124 | **-1.07** | GPR124 | **1.48** | HP | TargetScan |
| mmu-mir-124 | **-1.07** | HIC1 | **1.67** | HP | TargetScan |
| mmu-mir-124 | **-1.07** | NID1 | **1.02** | EO | TargetScan,miRecords |
| mmu-mir-124 | **-1.07** | PRRX1 | **1.90** | HP | TargetScan |
| mmu-mir-124 | **-1.07** | ROR2 | **1.05** | HP | TargetScan |
| mmu-mir-124 | **-1.07** | SEMA5A | **1.31** | HP | TargetScan |
| mmu-mir-124 | **-1.07** | SNAI2 | **1.29** | EO | TarBase,TargetScan,miRecords |
| mmu-mir-124 | **-1.07** | TMEM26 | **1.03** | HP | TargetScan |
| hsa-miR-363 | **-1.09** | ACTC1 | **2.93** | HP | TargetScan |
| hsa-miR-363 | **-1.09** | ADAMTS9 | **1.90** | HP | TargetScan |
| hsa-miR-363 | **-1.09** | ARHGAP29 | **1.08** | HP | TargetScan |
| hsa-miR-363 | **-1.09** | COL1A2 | **1.26** | HP | TargetScan |
| hsa-miR-363 | **-1.09** | DKK3 | **1.05** | HP | TargetScan |
| hsa-miR-363 | **-1.09** | GPR124 | **1.48** | HP | TargetScan |
| hsa-miR-363 | **-1.09** | HMGA2 | **1.41** | HP | TargetScan |
| hsa-miR-363 | **-1.09** | ITGA5 | **1.05** | EO | TargetScan,miRecords |
| hsa-miR-363 | **-1.09** | LHFPL2 | **1.15** | HP | TargetScan |
| hsa-miR-363 | **-1.09** | NEFM | **1.24** | HP | TargetScan |
| hsa-miR-363 | **-1.09** | NRK | **2.06** | HP | TargetScan |
| hsa-miR-363 | **-1.09** | PDE8A | **1.27** | HP | TargetScan |
| hsa-miR-363 | **-1.09** | STARD13 | **1.21** | HP | TargetScan |
| hsa-miR-363 | **-1.09** | TCFL5 | **1.82** | HP | TargetScan |
| hsa-miR-363 | **-1.09** | TWIST1 | **1.12** | HP | TargetScan |
| hsa-miR-363 | **-1.09** | VCL | **1.09** | HP | TargetScan |
| hsa-miR-595 | **-1.10** | ERBB3 | **1.62** | HP | TargetScan |
| hsa-miR-595 | **-1.10** | IGF1 | **2.53** | HP | TargetScan |
| mmu-miR-342-5p | **-1.11** | FRZB | **1.03** | HP | TargetScan |
| mmu-miR-342-5p | **-1.11** | IGF2 | **1.87** | HP | TargetScan |
| mmu-miR-326 | **-1.11** | EGFLAM | **1.36** | HP | TargetScan |
| mmu-miR-326 | **-1.11** | FZD4 | **1.17** | HP | TargetScan |
| mmu-miR-326 | **-1.11** | ITGA5 | **1.05** | HP | TargetScan |
| mmu-miR-326 | **-1.11** | NTN1 | **1.46** | HP | TargetScan |
| mmu-miR-326 | **-1.11** | SMOC2 | **1.14** | HP | TargetScan |
| mmu-miR-326 | **-1.11** | TAGLN2 | **1.41** | HP | TargetScan |
| hsa-miR-935 | **-1.17** | F11R | **1.16** | HP | TargetScan |
| mmu-miR-501-5p | **-1.22** | AHR | **1.20** | HP | TargetScan |
| mmu-miR-501-5p | **-1.22** | C7orf58 | **1.19** | HP | TargetScan |
| mmu-miR-501-5p | **-1.22** | HOXB7 | **1.13** | HP | TargetScan |
| mmu-miR-501-5p | **-1.22** | LPAR1 | **1.65** | HP | TargetScan |
| mmu-miR-501-5p | **-1.22** | LRIG3 | **1.43** | HP | TargetScan |
| mmu-miR-210 | **-1.25** | IGF2 | **1.87** | HP | TargetScan |
| mmu-miR-129-5p | **-1.48** | BNC2 | **1.00** | HP | TargetScan |
| mmu-miR-129-5p | **-1.48** | COL1A1 | **1.09** | HP | TargetScan |
| mmu-miR-129-5p | **-1.48** | DKK2 | **1.32** | HP | TargetScan |
| mmu-miR-129-5p | **-1.48** | DLC1 | **1.27** | HP | TargetScan |
| mmu-miR-129-5p | **-1.48** | EMP1 | **1.55** | HP | TargetScan |
| mmu-miR-129-5p | **-1.48** | HOXB7 | **1.13** | HP | TargetScan |
| mmu-miR-129-5p | **-1.48** | IGF1 | **2.53** | HP | TargetScan |
| mmu-miR-129-5p | **-1.48** | LAMA4 | **1.76** | HP | TargetScan |
| mmu-miR-129-5p | **-1.48** | LPAR4 | **1.14** | HP | TargetScan |
| mmu-miR-129-5p | **-1.48** | LRIG3 | **1.43** | HP | TargetScan |
| mmu-miR-129-5p | **-1.48** | ROR1 | **1.48** | HP | TargetScan |
| mmu-miR-129-5p | **-1.48** | S1PR3 | **1.64** | HP | TargetScan |
| mmu-miR-129-5p | **-1.48** | SEMA3D | **1.13** | HP | TargetScan |
| mmu-miR-129-5p | **-1.48** | SLIT3 | **1.13** | HP | TargetScan |
| mmu-miR-153 | **-1.51** | EGFLAM | **1.36** | HP | TargetScan |
| mmu-miR-153 | **-1.51** | GPR124 | **1.48** | HP | TargetScan |
| mmu-miR-153 | **-1.51** | PTCH1 | **1.04** | HP | TargetScan |
| mmu-miR-153 | **-1.51** | TBX2 | **1.35** | HP | TargetScan |
| mmu-miR-153 | **-1.51** | TGFBR2 | **1.28** | HP | TargetScan |
| hsa-miR-486-3p | **-1.65** | FAM101B | **1.15** | HP | TargetScan |
| hsa-miR-486-3p | **-1.65** | PDGFRB | **1.10** | HP | TargetScan |
| hsa-miR-486-3p | **-1.65** | S100A10 | **1.96** | HP | TargetScan |
| mmu-miR-449a | **-1.72** | ARHGDIB | **1.12** | HP | TargetScan |
| mmu-miR-449a | **-1.72** | BNC2 | **1.00** | HP | TargetScan |
| mmu-miR-449a | **-1.72** | CAPN6 | **1.70** | HP | TargetScan |
| mmu-miR-449a | **-1.72** | COL12A1 | **1.25** | HP | TargetScan |
| mmu-miR-449a | **-1.72** | IGFBP3 | **1.38** | HP | TargetScan |
| mmu-miR-449a | **-1.72** | PDGFRA | **1.54** | HP | TargetScan |
| mmu-miR-449a | **-1.72** | VCL | **1.09** | HP | TargetScan |
| mmu-mir-135a | **-1.75** | EDNRA | **2.22** | HP | TargetScan |
| mmu-mir-135a | **-1.75** | NEFM | **1.24** | HP | TargetScan |
| mmu-mir-135a | **-1.75** | NET1 | **1.07** | HP | TargetScan |
| mmu-mir-135a | **-1.75** | PLAGL1 | **1.01** | HP | TargetScan |
| mmu-mir-135a | **-1.75** | TGFBR2 | **1.28** | HP | TargetScan |
| mmu-miR-129-3p | **-1.81** | LHFPL2 | **1.15** | HP | TargetScan |
| hsa-miR-1251 | **-1.85** | IGF1 | **2.53** | HP | TargetScan |
| mmu-miR-302d | **-1.91** | AGTR2 | **1.26** | HP | TargetScan |
| mmu-miR-302d | **-1.91** | AHR | **1.20** | HP | TargetScan |
| mmu-miR-302d | **-1.91** | ARHGAP29 | **1.08** | HP | TargetScan |
| mmu-miR-302d | **-1.91** | BNC2 | **1.00** | HP | TargetScan |
| mmu-miR-302d | **-1.91** | CYBRD1 | **1.21** | HP | TargetScan |
| mmu-miR-302d | **-1.91** | ERBB3 | **1.62** | HP | TargetScan |
| mmu-miR-302d | **-1.91** | HOXB3 | **2.59** | HP | TargetScan |
| mmu-miR-302d | **-1.91** | HOXD8 | **1.36** | HP | TargetScan |
| mmu-miR-302d | **-1.91** | PDE8A | **1.27** | HP | TargetScan |
| mmu-miR-302d | **-1.91** | PRRX1 | **1.90** | HP | TargetScan |
| mmu-miR-302d | **-1.91** | SLC40A1 | **1.54** | HP | TargetScan |
| mmu-miR-302d | **-1.91** | TGFBR2 | **1.28** | HP | TargetScan |
| mmu-miR-302d | **-1.91** | TIMP3 | **1.29** | HP | TargetScan |
| mmu-miR-137 | **-1.93** | AHR | **1.20** | HP | TargetScan |
| mmu-miR-137 | **-1.93** | FBXL7 | **1.07** | HP | TargetScan |
| mmu-miR-137 | **-1.93** | HIC1 | **1.67** | HP | TargetScan |
| mmu-miR-137 | **-1.93** | ITGA5 | **1.05** | HP | TargetScan |
| mmu-miR-137 | **-1.93** | ITGA9 | **1.23** | HP | TargetScan |
| mmu-miR-137 | **-1.93** | KDELR3 | **1.16** | HP | TargetScan |
| mmu-miR-137 | **-1.93** | LHFPL2 | **1.15** | HP | TargetScan |
| mmu-miR-137 | **-1.93** | PDGFRA | **1.54** | HP | TargetScan |
| mmu-miR-137 | **-1.93** | PDLIM3 | **1.06** | HP | TargetScan |
| mmu-miR-137 | **-1.93** | SULF1 | **1.13** | HP | TargetScan |
| mmu-miR-137 | **-1.93** | TBX15 | **1.55** | HP | TargetScan |
| mmu-miR-137 | **-1.93** | TNFAIP6 | **1.51** | HP | TargetScan |
| mmu-miR-137 | **-1.93** | TWIST1 | **1.12** | HP | TargetScan |
| mmu-miR-491 | **-1.98** | BNC2 | **1.00** | HP | TargetScan |
| mmu-miR-491 | **-1.98** | EHD2 | **1.04** | HP | TargetScan |
| mmu-miR-491 | **-1.98** | IGF2 | **1.87** | HP | TargetScan |
| mmu-miR-491 | **-1.98** | PDGFRA | **1.54** | HP | TargetScan |
| mmu-miR-383 | **-2.05** | TMEM26 | **1.03** | HP | TargetScan |
| mmu-miR-199a-3p | **3.47** | SLC7A11 | **-1.12** | HP | TargetScan |
| mmu-miR-143 | **3.56** | SLC7A11 | **-1.12** | HP | TargetScan |
| mmu-miR-216b | **1.04** | GAD1 | **-1.47** | HP | TargetScan |
| mmu-miR-216b | **1.04** | PTCHD1 | **-1.07** | HP | TargetScan |
| mmu-miR-216b | **1.04** | SLC27A2 | **-1.32** | HP | TargetScan |
| mmu-miR-216b | **1.04** | SPHKAP | **-2.06** | HP | TargetScan |
| mmu-miR-216b | **1.04** | UNC5D | **-1.61** | HP | TargetScan |
| mmu-miR-216b | **1.04** | ZIC5 | **-1.42** | HP | TargetScan |
| mmu-miR-196a | **1.13** | CPM | **-1.36** | HP | TargetScan |
| mmu-miR-196a | **1.13** | LRP1B | **-1.34** | HP | TargetScan |
| mmu-miR-196a | **1.13** | RASGRP1 | **-1.76** | HP | TargetScan |
| mmu-miR-196a | **1.13** | RSPO2 | **-2.46** | HP | TargetScan |
| mmu-miR-196a | **1.13** | TRHDE | **-1.04** | HP | TargetScan |
| mmu-miR-196a | **1.13** | VSNL1 | **-1.38** | HP | TargetScan |
| hsa-miR-612 | **1.16** | FAM163A | **-1.47** | HP | TargetScan |
| hsa-miR-612 | **1.16** | SPHKAP | **-2.06** | HP | TargetScan |
| mmu-miR-351 | **1.25** | CBLN2 | **-2.17** | EO | miRecords |
| mmu-miR-351 | **1.25** | CPM | **-1.36** | HP | TargetScan |
| hsa-miR-217 | **1.27** | SLC7A11 | **-1.12** | HP | TargetScan |
| hsa-miR-217 | **1.27** | VSNL1 | **-1.38** | HP | TargetScan |
| mmu-miR-322 | **1.29** | EN2 | **-1.18** | HP | TargetScan |
| mmu-miR-322 | **1.29** | LRP1B | **-1.34** | HP | TargetScan |
| mmu-miR-322 | **1.29** | OMG | **-1.27** | HP | TargetScan |
| mmu-miR-322 | **1.29** | OTX1 | **-1.60** | HP | TargetScan |
| mmu-miR-322 | **1.29** | RSPO3 | **-1.65** | HP | TargetScan |
| mmu-miR-322 | **1.29** | SPOCK3 | **-1.11** | HP | TargetScan |
| mmu-miR-322 | **1.29** | TFAP2D | **-1.36** | HP | TargetScan |
| mmu-miR-574-5p | **1.42** | SPOCK3 | **-1.11** | HP | TargetScan |
| mmu-miR-133a | **1.56** | GLRA2 | **-1.50** | HP | TargetScan |
| mmu-miR-133a | **1.56** | GRM5 | **-1.47** | HP | TargetScan |
| mmu-miR-133a | **1.56** | PGAP1 | **-1.24** | HP | TargetScan |
| mmu-miR-133a | **1.56** | TFAP2D | **-1.36** | HP | TargetScan |
| mmu-miR-133a | **1.56** | TRHDE | **-1.04** | HP | TargetScan |
| mmu-miR-133a | **1.56** | ZIC3 | **-1.21** | HP | TargetScan |
| mmu-miR-10a | **4.58** | GABRB2 | **-1.06** | HP | TargetScan |
| mmu-miR-10a | **4.58** | UNC5D | **-1.61** | HP | TargetScan |
| mmu-miR-669d | **1.77** | RSPO3 | **-1.65** | HP | TargetScan |
| hsa-miR-422a | **1.82** | ZFPM2 | **-1.21** | HP | TargetScan |
| mmu-miR-297c | **2.02** | CYP4F2 | **-1.17** | HP | TargetScan |
| mmu-miR-297c | **2.02** | KCND2 | **-1.71** | HP | TargetScan |
| mmu-miR-297c | **2.02** | NKAIN2 | **-1.11** | HP | TargetScan |
| mmu-miR-297c | **2.02** | TFAP2B | **-1.39** | HP | TargetScan |
| hsa-miR-595 | **2.02** | GABRA1 | **-1.67** | HP | TargetScan |
| hsa-miR-595 | **2.02** | PGAP1 | **-1.24** | HP | TargetScan |
| hsa-miR-1322 | **2.08** | GRM1 | **-1.42** | HP | TargetScan |
| hsa-miR-1322 | **2.08** | UNC5D | **-1.61** | HP | TargetScan |
| mmu-miR-214 | **3.12** | C1orf110 | **-1.35** | HP | TargetScan |
| mmu-miR-214 | **3.12** | TFAP2D | **-1.36** | HP | TargetScan |
| mmu-miR-199a-5p | **3.29** | OTX1 | **-1.60** | HP | TargetScan |
| mmu-miR-206 | **6.64** | ANO1 | **-1.07** | HP | TargetScan |
| mmu-miR-206 | **6.64** | OTX2 | **-1.24** | HP | TargetScan |
| mmu-miR-206 | **6.64** | RSPO3 | **-1.65** | HP | TargetScan |
| mmu-miR-206 | **6.64** | SLC7A11 | **-1.12** | HP | TargetScan |
| mmu-miR-206 | **6.64** | ZIC4 | **-1.88** | HP | TargetScan |

* HP – high predicted, EO – experimentally observed
